# Neoantigen-based immunotherapy: advancing precision medicine in cancer and glioblastoma treatment through discovery and innovation

**DOI:** 10.37349/etat.2025.1002313

**Published:** 2025-04-27

**Authors:** Moawiah M Naffaa, Ola A Al-Ewaidat, Sopiko Gogia, Valiko Begiashvili

**Affiliations:** The First Clinical Medical College of Lanzhou University, China; ^1^Department of Psychology and Neuroscience, Duke University, Durham, NC 27708, USA; ^2^Department of Cell Biology, Duke University School of Medicine, Durham, NC 27710, USA; ^3^Department of Internal Medicine, Ascension Saint Francis Hospital, Evanston, IL 60202, USA; ^4^Department of Internal Medicine, University of Kansas Medical Center, Kansas City, KS 66103, USA

**Keywords:** Neoantigen-based immunotherapy, precision medicine, cancer, glioblastoma, CAR-T, tumor-infiltrating lymphocyte therapy, tumor microenvironment, AI-driven tools

## Abstract

Neoantigen-based immunotherapy has emerged as a transformative approach in cancer treatment, offering precision medicine strategies that target tumor-specific antigens derived from genetic, transcriptomic, and proteomic alterations unique to cancer cells. These neoantigens serve as highly specific targets for personalized therapies, promising more effective and tailored treatments. The aim of this article is to explore the advances in neoantigen-based therapies, highlighting successful treatments such as vaccines, tumor-infiltrating lymphocyte (TIL) therapy, T-cell receptor-engineered T cells therapy (TCR-T), and chimeric antigen receptor T cells therapy (CAR-T), particularly in cancer types like glioblastoma (GBM). Advances in technologies such as next-generation sequencing, RNA-based platforms, and CRISPR gene editing have accelerated the identification and validation of neoantigens, moving them closer to clinical application. Despite promising results, challenges such as tumor heterogeneity, immune evasion, and resistance mechanisms persist. The integration of AI-driven tools and multi-omic data has refined neoantigen discovery, while combination therapies are being developed to address issues like immune suppression and scalability. Additionally, the article discusses the ongoing development of personalized immunotherapies targeting tumor mutations, emphasizing the need for continued collaboration between computational and experimental approaches. Ultimately, the integration of cutting-edge technologies in neoantigen research holds the potential to revolutionize cancer care, offering hope for more effective and targeted treatments.

## Introduction

The discovery of eukaryotic split genes, with non-coding intronic sequences interspersed within protein-coding regions, revolutionized our understanding of gene structure and regulation. This breakthrough illuminated the process of RNA splicing, where introns are excised from pre-messenger RNA (pre-mRNA) by the spliceosome, a ribonucleoprotein complex that ensures the correct ligation of exons to form mature mRNA for translation [1, 2]. Splicing, which occurs in approximately 94% of human genes, is a ubiquitous and essential process in higher eukaryotes, playing a pivotal role in gene expression. Notably, a significant portion of these genes undergoes alternative splicing, generating diverse mRNA isoforms that enable a single gene to produce multiple protein variants [3–5]. This diversification is essential for cellular differentiation, development, and homeostasis. Aberrant splicing, however, can lead to the production of dysfunctional protein isoforms, contributing to various pathological conditions, including cancer and neurodegenerative diseases [6–8]. Furthermore, alternative splicing plays a crucial role in the presentation of neoantigens, which are tumor-specific antigens (TSAs) formed due to genetic mutations and splicing alterations. These neoantigens have emerged as key targets for cancer immunotherapies, highlighting the significance of understanding splicing mechanisms in the development of novel therapeutic strategies.

In cancer biology, the concept of TSAs, or neoantigens, has emerged as a transformative approach in immunotherapy [9, 10]. Neoantigens arise from somatic mutations and are presented on tumor cell surfaces by major histocompatibility complex (MHC) molecules, enabling cytotoxic T lymphocytes (CTLs) to target and eliminate tumor cells [9, 11, 12]. The identification of neoantigens has catalyzed the development of cancer vaccines and adoptive T cell therapies, all of which amplify tumor-specific immune responses [9, 13, 14]. Notably, neoantigens are exclusive to tumor cells, minimizing off-target effects and enabling personalized therapeutic approaches.

While most neoantigen research has focused on mutations, recent studies have highlighted a novel source: aberrant RNA splicing. Dysregulated splicing can produce cryptic exons or exon-skipping events, leading to the generation of tumor-specific splicing isoforms that may serve as immunotherapeutic targets [3]. Advances in next-generation sequencing (NGS) and RNA-seq technologies have facilitated the profiling of cancer-specific splicing landscapes, uncovering new neoepitopes [15, 16]. Additionally, predictive bioinformatics tools that assess MHC-binding affinity and immunogenicity further expand the potential pool of actionable targets [17, 18].

Glioblastomas (GBMs) exemplify the need for innovative therapies. Despite multimodal treatments, including surgery, radiotherapy, and chemotherapy with temozolomide, median survival remains limited to 15 months [19, 20]. FDA-approved therapies such as bevacizumab and tumor-treating fields (TTF) offer minimal survival benefits, and immunotherapeutic agents like nivolumab and EGFRvIII vaccines have failed to achieve efficacy in phase 3 trials. GBM’s inherent heterogeneity, infiltrative growth, and immunosuppressive microenvironment contribute to its resistance to conventional treatments [21, 22].

Neoantigen-based personalized peptide vaccines offer a promising avenue for GBM treatment [9, 23]. By utilizing NGS to identify tumor-specific mutations and splicing variants, these vaccines target unique neoepitopes, generating robust and specific immune responses. Clinical trials targeting the H3K27M mutation in diffuse midline gliomas have demonstrated safety and immunogenicity, underscoring the potential of personalized immunotherapy for GBM management [24, 25]. This strategy represents a critical step in addressing the challenges posed by this devastating malignancy.

This article explores the transformative potential of neoantigen-based immunotherapy, a precision oncology approach that targets TSAs arising from genetic, transcriptomic, and proteomic alterations. It examines various therapeutic modalities, including neoantigen vaccines, tumor-infiltrating lymphocyte (TIL) therapy, T-cell receptor-engineered T cells therapy (TCR-T), and chimeric antigen receptor T cells therapy (CAR-T), all of which show promise in treating GBM. The article also highlights how advances in NGS, RNA-based platforms, and CRISPR gene-editing technologies have accelerated neoantigen discovery, facilitating the development of highly personalized treatments. However, clinical application remains challenging due to factors such as tumor heterogeneity, immune evasion, and therapeutic resistance. To overcome these obstacles, the article underscores the need for an integrated approach that combines multi-omic data analysis, AI-driven predictive modeling, and collaborative efforts between researchers and clinicians to optimize neoantigen-based immunotherapies, paving the way for more effective and durable cancer treatments.

## Advances in neoantigen research: from early insights to clinical applications in targeted cancer immunotherapy

Neoantigen research has significantly advanced our understanding of tumor immunology and has been crucial in the development of targeted cancer therapies. Neoantigens are tumor-specific proteins that arise from somatic mutations in cancer cells, making them highly specific and effective targets for immune system recognition. The identification and validation of these neoantigens have revolutionized cancer immunotherapy by opening up new possibilities for personalized treatments [10, 26].

The exploration of neoantigens dates back to foundational studies in the mid-20th century, which demonstrated the immune system’s ability to recognize tumor cells as foreign [27, 28]. These early insights set the stage for further discoveries, including the recognition of T cell-mediated immune responses against neoantigens in both murine and human tumor models, validating their immunogenic potential [13, 29].

The technological breakthroughs of the early 2000s, such as NGS and flow cytometry, played a pivotal role in advancing neoantigen research. These tools allowed for high-throughput identification and validation of neoantigens, deepening our understanding of tumor immunology and T cell responses [30, 31].

Key milestones in neoantigen research are summarized in Table 1, highlighting how the field has evolved from initial discoveries to the application of personalized neoantigen vaccines and adoptive cell therapies (ACTs). The progress made in identifying and targeting TSAs demonstrates the transformative potential of these.

**Table 1:** Timeline of key milestones and technological advances in neoantigen-based cancer immunotherapy

**Year**	**Milestone**	**Impact on cancer research**	**Key technologies**	**Therapeutic applications**	**References**
1950s	Immune recognition of carcinogen-induced tumors	Role of immune system in tumor detection	None	Early understanding	[32, 33]
1980s	T cell recognition of neoantigens in mice	Cellular immune mechanisms	Murine models	None	[14, 34]
1990s	Confirmed neoantigen reactivity in mice	Evidence for tumor-specific immune responses	Murine models	None	[35, 36]
1990s	Neoantigen reactivity in human cancers	Broadened immune detection	Human tumor models	None	[13]
2000s	Neoantigen reactivity in CD4+ T cells	Identified immune cell responses	Flow cytometry	None	[37]
2000s	Identified neoantigen-reactive T cells in melanoma patients	Enhanced clinical relevance	Flow cytometry	None	[38, 39]
2000s	Neoantigen reactivity over shared antigens	Clarified neoantigen advantage	Human tumor models	None	[40]
2010s	NGS for immunogenic neoantigen identification	Accelerated antigen discovery	NGS	None	[41, 42]
2010s	NGS for neoantigen-reactive T cells in melanoma	Mapped T cell specificity	NGS, flow cytometry	None	[43, 44]
2010s	Neoantigen-based adoptive cell therapy in GI cancer	First clinical application	NGS, adoptive cell therapy	GI cancer treatment	[45, 46]
2010s	Personalized neoantigen vaccines for melanoma	Pioneered patient-specific vaccines	NGS, peptide synthesis	Melanoma treatment	[47, 48]
2010s	KRAS neoantigen adoptive cell therapy in colorectal cancer	Targeted oncogene mutations	NGS, adoptive cell therapy	Colorectal cancer treatment	[49, 50]
2010s	Personalized neoantigen peptide and RNA vaccines	Advanced specificity and delivery methods	NGS, RNA technology	Multiple tumor types	[51, 52]

NGS: next-generation sequencing; GI: gastrointestinal

Recent advancements in technologies, such as NGS and RNA-based platforms, have greatly accelerated the clinical translation of neoantigen research, enhancing the specificity and efficacy of therapeutic strategies [31, 53]. Ongoing innovations continue to refine neoantigen-based immunotherapies by improving immune responses while reducing off-target effects.

Looking ahead, the integration of cutting-edge technologies like CRISPR and AI may revolutionize the discovery and validation of neoantigens. These advancements promise to further enhance the effectiveness of cancer treatments, broadening the applicability of neoantigen-based therapies across various cancer types.

## Mechanisms driving neoantigen diversity: genetic, transcriptomic, and proteomic contributions

Neoantigen generation results from a multifaceted interplay of genetic, transcriptomic, and proteomic mechanisms, all of which contribute to the diversity of the antigenic landscape. These molecular alterations can profoundly affect the immune system’s ability to recognize cancer cells by presenting novel peptide sequences that are not found in normal, healthy tissues [54, 55].

At the genomic level, various mutations, such as single nucleotide variants (SNVs), insertions and deletions (INDELs), and gene fusions, disrupt the normal coding sequences of genes (Table 2). These alterations can lead to the creation of novel open reading frames (ORFs) and chimeric proteins that may possess antigenic properties, making them recognizable by the immune system [56, 57]. Additionally, the integration of viral sequences or the reactivation of endogenous retroviruses (ERVs) further contributes to the diversity of the neoantigen repertoire by introducing foreign protein-coding regions into the genome [58, 59]. These changes can be particularly impactful in cancer, where such genomic alterations can create unique targets for immune recognition.

**Table 2:** Classification of neoantigen sources in cancer: genomic, transcriptomic, and proteomic alterations

**Category**	**Mechanism**	**Description**	**Neoantigen impact**	**References**
Genomic mutations	Single nucleotide variants (SNVs)	A single-base substitution causes amino acid changes.	Alters the coding sequence, generating novel peptides.	[60]
	Insertions and deletions (INDELs)	Small insertions or deletions lead to frameshift mutations.	Frameshift mutations alter amino acid sequences and generate novel open reading frames (ORFs).	[61, 62]
	Gene fusions	Fusion of two genes creating a chimeric protein.	Generates unique antigenic epitopes due to the formation of hybrid peptides.	[63]
	Viral sequences and endogenous retroviruses (ERVs)	Integration/reactivation of viral sequences in the genome.	Introduces foreign protein-coding sequences, producing immunogenic peptides.	[64, 65]
Transcriptomic variants	Constitutive splicing	Standard intron removal and exon joining.	Preserves the normal coding sequence, minimal neoantigen impact.	[66, 67]
	Exon skipping/inclusion	Variable inclusion or exclusion of exons.	Modifies the protein structure, potentially generating novel peptide regions.	[68, 69]
	Alternative 5' and 3' splice sites	Variations in donor and acceptor splice site selection.	Shifts exon boundaries, altering peptide sequence diversity.	[70, 71]
	Intron retention	Failure to remove an intron during splicing.	The translation of non-coding regions can introduce premature stop codons and novel antigenic sites.	[72, 73]
	Mutually exclusive exons	Expression of one exon while excluding another.	Generates diverse protein isoforms with unique antigenic determinants.	[74, 75]
	Exitrons	Hybrid exonic-intronic regions where introns are partially retained.	Alters the final protein product, increasing peptide diversity.	[76, 77]
	Adenosine-to-inosine (A-to-I) RNA editing	Post-transcriptional modification converting adenosine to inosine.	Alters codons without changing the underlying genomic sequence, leading to novel peptide generation.	[78, 79]
Proteomic variants	ORFs	Translation initiated from previously untranslated regions.	Produces peptides not expressed under normal conditions, expanding neoantigen diversity.	[80, 81]
	Coding long non-coding RNAs (lncRNAs)	Some lncRNAs can generate small peptides.	Generates small immunogenic peptides despite being non-coding.	[82, 83]
	Defective translation	Translational errors such as frameshifts or premature termination.	Produces aberrant protein fragments, expanding the neoantigen repertoire.	[84]
	Alternative start sites	Translation initiated from non-canonical codons (CUG, AGG, AUA).	Generates alternative protein isoforms with novel N-terminal sequences.	[85, 86]
	Post-translational modifications	Modifications like phosphorylation, glycosylation, ubiquitination.	Alters peptide structure and may expose hidden epitopes for immune recognition.	[87, 88]

The transcriptomic landscape also plays a crucial role in expanding neoantigen diversity. Alternative mRNA processing events, such as exon skipping, alternative splice site selection, and intron retention, can modify the protein-coding potential of genes (Table 2). These variations can lead to the translation of modified or truncated protein products, which may differ significantly from the normal protein isoform [89–91]. In addition, mechanisms like mutually exclusive exon expression and the partial retention of exitrons further contribute to the diversity of protein isoforms that can be produced from a single gene [75]. Moreover, post-transcriptional modifications, such as adenosine-to-inosine (A-to-I) RNA editing, introduce codon changes that alter the sequence of the resulting peptides without modifying the underlying DNA sequence. This adds another layer of potential neoantigen generation from modified RNA transcripts [92, 93].

At the proteomic level, the generation of neoantigens is further enhanced by variations in translation and post-translational modifications (Table 2). ORFs can emerge from untranslated regions of the genome, and small peptides derived from long non-coding RNAs (lncRNAs) may also generate unique antigenic peptides [94, 95]. Translation defects, such as premature termination, frameshift mutations, or the use of non-canonical start sites, also contribute to antigenic diversity by producing altered peptide sequences that are not present in healthy tissues [96]. Additionally, post-translational modifications, such as phosphorylation, glycosylation, and ubiquitination, can expose previously hidden epitopes or alter peptide structures in ways that make them more immunologically recognizable [97, 98].

A comprehensive summary of the genetic, transcriptomic, and proteomic mechanisms that contribute to neoantigen diversity is provided in Table 2.

## Neoantigen generation and treatment modalities in cancer immunotherapy

The development of effective cancer immunotherapies relies heavily on the identification and targeting of tumor-specific neoantigens. Neoantigens, which arise from genetic and molecular alterations unique to cancer cells, play a critical role in immune recognition and the design of personalized treatments [26, 42, 99]. Understanding how various therapeutic strategies influence neoantigen generation and presentation is essential for optimizing immunotherapy efficacy.

**Figure 1:**
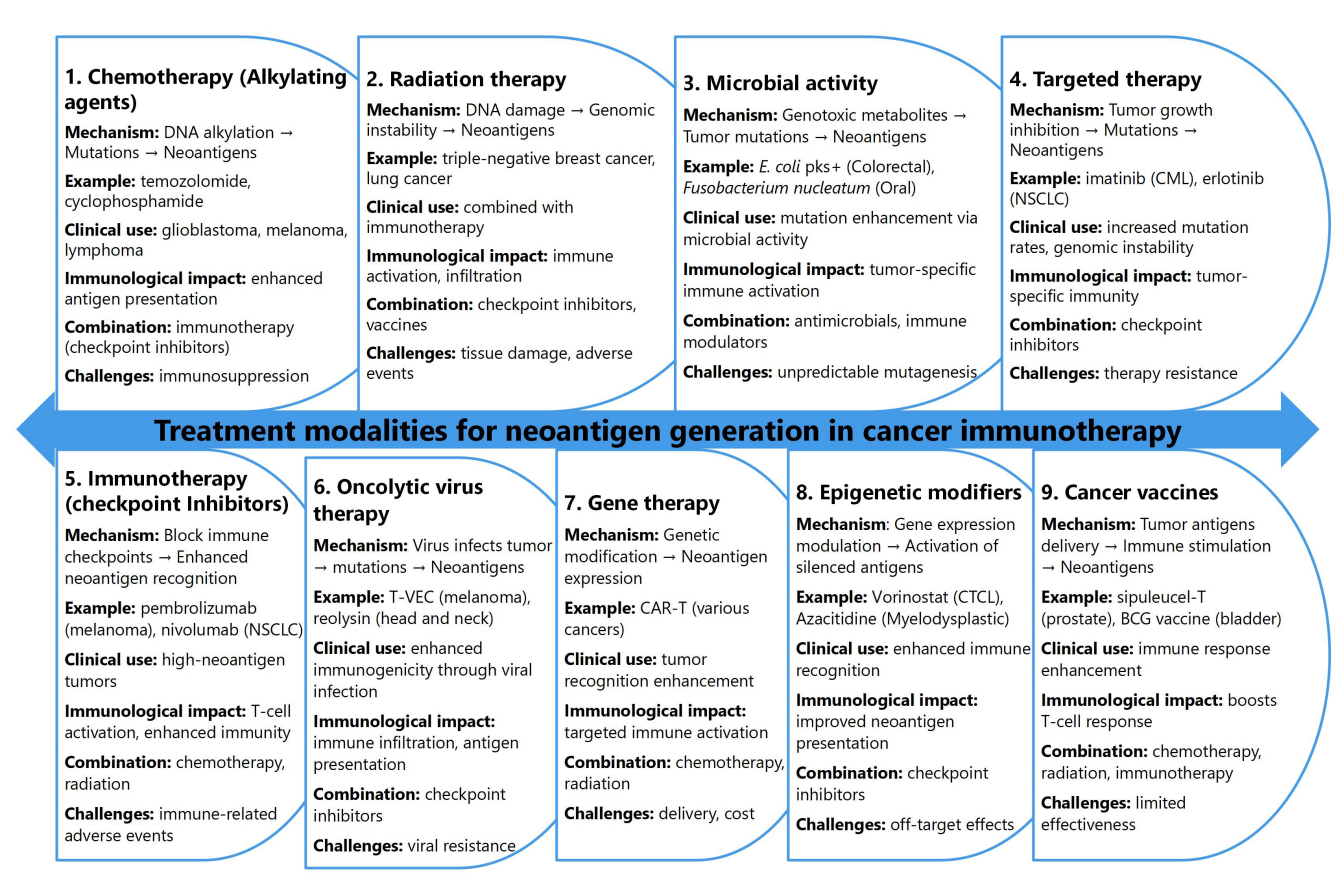
Treatment modalities and their role in neoantigen generation for cancer immunotherapy

A comprehensive overview of various treatment modalities and their roles in neoantigen generation within the context of cancer immunotherapy are discussed in Figure 1. Each therapeutic approach affects neoantigen formation through distinct biological mechanisms, with implications for immune response modulation, clinical application, and combination therapy potential.

Chemotherapy, particularly alkylating agents such as temozolomide and cyclophosphamide, induces DNA alkylation that causes direct strand damage, leading to point mutations and small insertions or deletions [100–102]. These genetic alterations give rise to novel antigenic peptides presented on MHC molecules, making tumor cells more immunogenic. For example, temozolomide treatment has been associated with enhanced neoantigen expression in GBM, particularly when paired with cancer vaccines [23]. However, chemotherapy poses challenges in predicting the extent of neoantigen generation and may cause immunosuppression, complicating its standalone efficacy [103, 104].

Radiation therapy also contributes significantly to neoantigen generation by causing DNA damage through direct ionization and reactive oxygen species (ROS) production. This damage induces genomic instability, leading to the formation of neoantigens from fragmented or mutated tumor DNA [105–107]. Preclinical studies in triple-negative breast cancer and non-small cell lung cancer (NSCLC) have demonstrated that radiation, when combined with immune checkpoint inhibitors such as anti-programmed death-1 (PD-1) inhibitors, enhances antitumor immunity by increasing immune cell infiltration [108, 109]. Nonetheless, radiation therapy carries the risk of localized tissue damage and immune-related adverse effects [110].

Microbial activity, specifically genotoxic microbial metabolites such as colibactin produced by *Escherichia coli*, can lead to tumor-specific mutations. Strains like pks+ *E. coli* induce double-strand breaks in host DNA, contributing to neoantigen formation in colorectal cancer [111, 112]. Similarly, *Fusobacterium nucleatum* and *Bacteroides fragilis* have been linked to mutagenic effects in colorectal and oral cancers [113, 114]. These microbial-induced neoantigens can promote tumor-specific immune responses, yet challenges remain in targeting specific microbial strains without disrupting the broader microbiome [115].

Targeted therapies, such as tyrosine kinase inhibitors (e.g., imatinib and erlotinib), interfere with signaling pathways involved in tumor growth and survival. These agents can induce mutations or unmask cryptic antigens within the tumor microenvironment (TME) [116, 117]. For instance, imatinib has been associated with increased mutation rates in chronic myelogenous leukemia, contributing to antigenic diversity [118]. However, the emergence of resistance and limited efficacy in tumors with low mutation rates restricts the widespread impact of targeted therapies on neoantigen generation [119].

Immunotherapy, particularly immune checkpoint inhibitors (e.g., pembrolizumab and nivolumab), plays a central role in restoring immune surveillance by blocking inhibitory proteins like PD-1 and CTLA-4 [120, 121]. These inhibitors amplify T-cell activity against tumor-specific neoantigens, especially in tumors with high mutational burdens. However, immune-related adverse events, such as colitis and pneumonitis [11, 122], pose clinical management challenges, necessitating careful patient selection.

Emerging therapeutic modalities, including oncolytic virus therapy and gene therapy, further expand the landscape of neoantigen generation. Oncolytic viruses like T-VEC and Reolysin selectively infect tumor cells, inducing direct lysis and the release of neoantigens [123, 124]. Additionally, viral infection can trigger immunogenic cell death, promoting immune system activation [125]. Gene therapy approaches, such as CAR-T cell therapy and oncolytic virus gene modification, enhance neoantigen presentation through engineered genetic alterations that promote novel antigen expression [126]. However, these advanced therapies face challenges like delivery efficiency, off-target effects, and high costs.

Epigenetic modifiers, including DNA methylation inhibitors and histone deacetylase inhibitors like vorinostat and azacitidine, represent another innovative strategy for neoantigen generation [127, 128]. By altering the epigenetic landscape, these agents can reactivate silenced tumor antigens and promote immune recognition [129, 130]. Nonetheless, concerns regarding off-target epigenetic changes and unpredictable long-term effects remain significant hurdles.

## Advancing cancer immunotherapy: the role of neoantigen-based approaches

### Neoantigen-based immunotherapies—personalized approaches and synergistic strategies in cancer treatment

**Figure 2:**
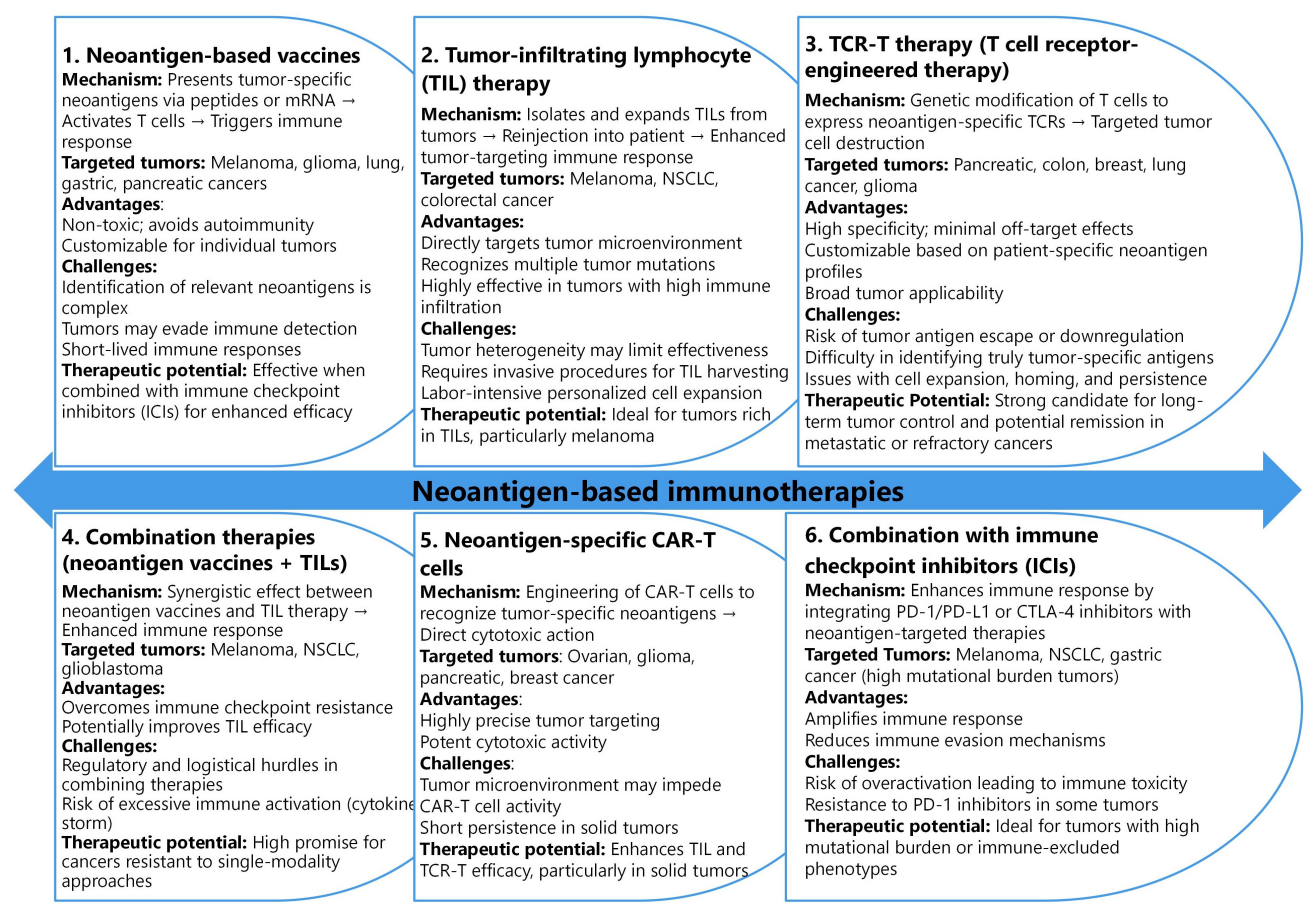
Comparative analysis of neoantigen-based immunotherapies: mechanisms, targeted tumors, and clinical potential

Neoantigen-based immunotherapies represent a promising frontier in cancer treatment, leveraging the body’s immune system to target TSAs [131]. These therapies encompass a variety of approaches, including vaccines, TIL therapy, TCR-T therapy, and CAR-T cells, each designed to stimulate or enhance immune responses against tumor neoantigens [132] (Figure 2). The breadth of neoantigen-targeted strategies underscores the potential for personalized cancer treatments [133, 134], emphasizing the importance of tailoring interventions to the unique antigenic landscape of individual tumors.

Neoantigen vaccines, for instance, aim to stimulate T cells by presenting neoantigen peptides or mRNA to the immune system, primarily targeting tumors such as melanoma, glioma, and lung cancer [131]. These vaccines offer advantages like non-toxicity and the ability to customize treatment for individual patients. However, challenges remain, including limited neoantigen identification and immune evasion by tumors. Despite these hurdles, studies have demonstrated the high therapeutic potential of neoantigen vaccines, particularly when combined with immune checkpoint inhibitors, leading to improved survival rates and tumor shrinkage [135, 136].

TIL therapy involves isolation, expansion, and reinfusion of TILs from the patient’s tumor to enhance antitumor activity. This approach has shown effectiveness in cancers with high TIL burden, such as melanoma and NSCLC [137, 138]. TIL therapy’s advantages include its ability to target multiple tumor mutations simultaneously and its direct engagement with the TME. However, invasive procedures for TIL harvesting and challenges related to tumor heterogeneity pose significant limitations [138]. Nonetheless, TIL therapy remains a powerful option, especially in immune-permissive tumors, with notable results in metastatic colorectal cancer [139, 140].

Combination therapies, including the use of neoantigen vaccines with TILs or immune checkpoint inhibitors, have gained attention for their synergistic effects in overcoming immune resistance [141]. As shown in Figure 2, combining these therapies can enhance the immune response by targeting multiple pathways simultaneously, particularly in cancers with high mutational burdens like melanoma and NSCLC [142, 143]. However, complex logistics, regulatory hurdles, and the risk of excessive immune activation remain barriers to widespread clinical adoption [144]. Still, the potential for achieving complete remission in refractory cancers underscores the promise of these multimodal strategies in advancing cancer immunotherapy.

### Comparative analysis of neoantigen-based therapeutic strategies in cancer immunotherapy

Neoantigen-based therapeutic strategies represent a transformative advancement in cancer immunotherapy. These neoantigens are absent in normal tissues, allowing for precise tumor targeting with minimal risk to healthy cells [133, 145]. By focusing on these highly specific antigens, neoantigen therapies aim to harness and amplify the immune system’s natural ability to recognize and destroy malignant cells, thereby reducing the likelihood of off-target cytotoxicity and enhancing the therapeutic index [146, 147]. This precision is particularly advantageous in cancers with high mutational burdens, where the diversity of neoantigens increases the likelihood of effective immune responses [11, 148].

The principal neoantigen-based strategies include TCR-T therapy, CAR-T cell therapy, bispecific antibodies, neoantigen vaccines, TIL therapy, oncolytic virus therapies, cancer peptide vaccines, dendritic cell (DC) vaccines, and personalized multi-epitope vaccines [149]. TCR-T therapy involves the genetic engineering of autologous T cells to express TCRs capable of recognizing neoantigens presented by MHC-I molecules on the tumor cell surface [150]. CAR-T therapy modifies T cells to express synthetic antigen-binding receptors that can directly recognize tumor surface antigens without the need for MHC presentation, broadening their applicability to tumors with low MHC expression [151]. Bispecific antibodies act as immune engagers by simultaneously binding TSAs and CD3 on T cells, physically linking effector cells to tumor cells for targeted cytotoxicity [152, 153]. Neoantigen vaccines, including RNA, DNA, peptide, and DC-based platforms, work by introducing TSAs into the host to prime and expand tumor-reactive T cells [131, 154]. TIL therapy involves isolating and expanding TILs directly from the patient’s tumor, which are then reinfused into the patient to boost anti-tumor immunity [140, 155, 156]. Oncolytic virus therapies use genetically modified viruses that selectively infect and lyse tumor cells while also stimulating a systemic immune response against neoantigens released during tumor cell lysis [126]. Cancer peptide vaccines use synthetic peptides corresponding to TSAs to stimulate T cell responses, while DC vaccines involve loading patient-derived DCs with tumor neoantigens ex vivo before reinfusion [157–159]. Personalized multi-epitope vaccines combine multiple tumor neoantigens tailored to an individual’s tumor mutation profile, maximizing immune coverage [160, 161].

**Figure 3:**
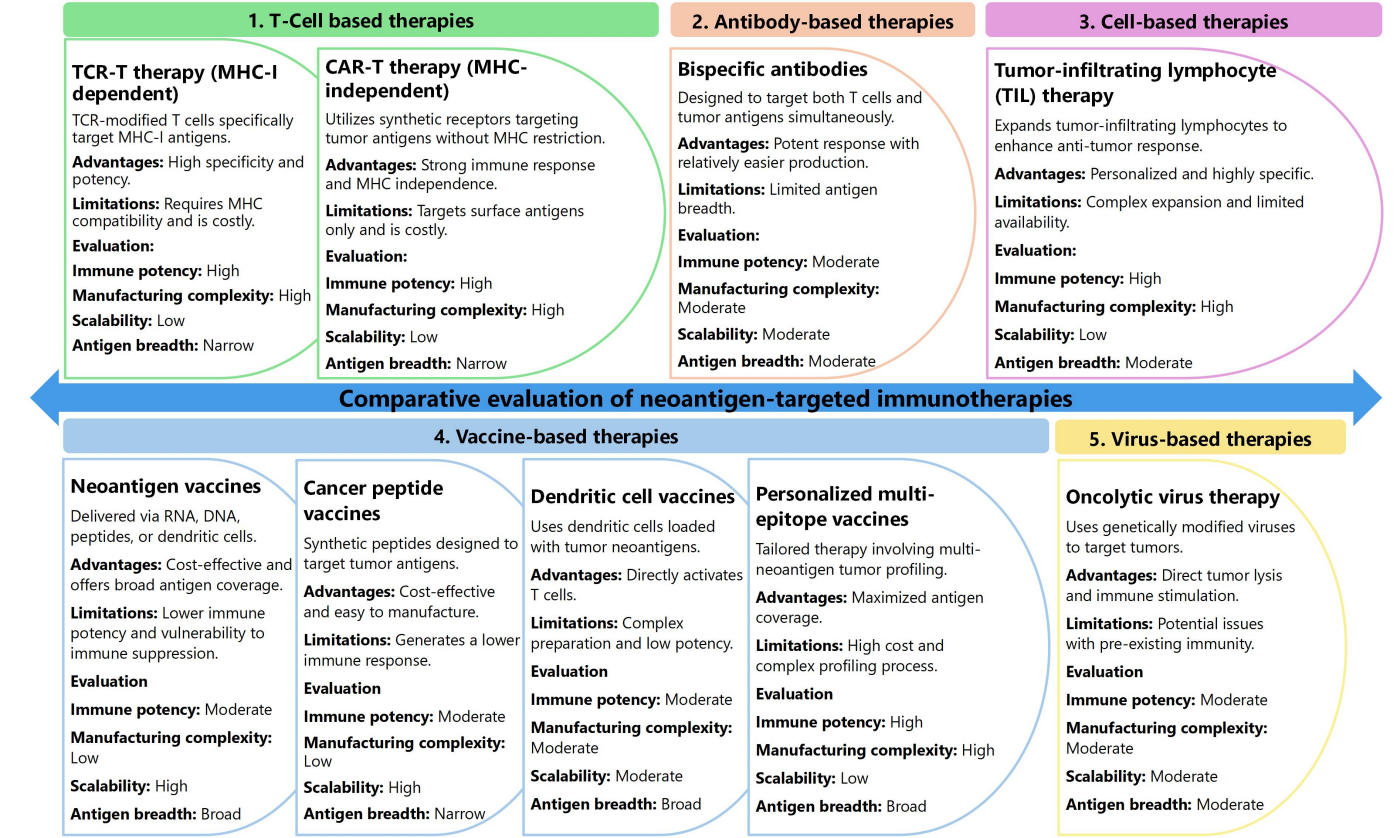
Comparative evaluation of neoantigen-targeted immunotherapies: key features, advantages, and clinical feasibility

A comparative analysis of these neoantigen-based therapeutic strategies, evaluating critical parameters such as immune response potency, manufacturing complexity, scalability, and antigen breadth, is presented in Figure 3. TCR-T and CAR-T therapies exhibit robust immune responses but face challenges due to high manufacturing complexity and limited scalability, as they require patient-specific modifications [162, 163]. Bispecific antibodies provide a balance of moderate potency and manageable manufacturing demands, making them versatile, though still constrained by limited antigen breadth [164]. Neoantigen vaccines, while highly scalable and capable of broad antigen coverage, often elicit weaker immune responses and struggle to overcome immune suppression in advanced malignancies [146]. TIL therapy offers a potent and personalized response but relies on the availability of tumor-infiltrating cells and involves complex ex vivo expansion protocols [156]. Oncolytic viruses provide a dual mechanism of direct tumor lysis and immune activation but are often limited by pre-existing immunity against the viral vector [165, 166]. Peptide and DC vaccines offer moderate scalability and are less complex to produce but face challenges in generating sustained immune responses [167, 168]. Personalized multi-epitope vaccines maximize antigen breadth but require highly individualized tumor profiling, increasing complexity and cost [134, 141]. This comparative framework aids in selecting the optimal strategy based on clinical context and disease stage.

### Revolutionizing cancer treatment through neoantigen-based therapeutics

The development of neoantigen-based therapeutic strategies has heralded a transformative era in cancer immunotherapy, capitalizing on TSAs derived from somatic mutations [99, 169]. These neoantigens, exclusive to malignant cells and absent in normal tissues, facilitate precise tumor targeting while minimizing collateral damage to healthy tissues [11, 131, 170]. The convergence of precision medicine and immunotherapeutic innovations positions neoantigen-based therapies as a cornerstone of personalized cancer treatment [103, 169]. Their potential is particularly compelling in patients with high mutational burdens, where the presence of diverse neoantigens drives robust immune responses [171].

Technological advancements have significantly enhanced neoantigen identification and engineering, ensuring higher precision and efficacy in therapeutic development. The integration of AI and high-throughput sequencing has revolutionized neoantigen discovery [172, 173]. AI-driven algorithms analyze extensive genomic and proteomic datasets, expediting the identification of TSAs and enabling tailored treatments for individual tumor profiles [174]. Simultaneously, CRISPR/Cas9 gene-editing technology has expanded the therapeutic horizon by optimizing immune cells to improve neoantigen recognition [175]. The engineering of potent TCR-T and CAR-T cell therapies using CRISPR has been particularly impactful, overcoming challenges such as immune evasion and resistance and broadening the applicability of neoantigen-based strategies across diverse cancer types [175].

A pivotal milestone in advancing neoantigen-based immunotherapies lies in the integration of multi-omic data, including genomics, transcriptomics, proteomics, and metabolomics [176]. This comprehensive approach deepens our understanding of tumor biology, enabling the identification of a personalized, exhaustive set of neoantigens [177, 178]. Multi-omic profiling facilitates the design of immunotherapies that address both well-characterized and novel neoantigens [179, 180]. The advent of personalized multi-epitope vaccines, enriched by insights from multi-omic analyses, has demonstrated enhanced antigen selection and broadened immune coverage [181, 182]. These precision-driven vaccines underscore the transformative potential of combining molecular-level insights with immunotherapeutic strategies.

Despite their transformative promise, neoantigen-based therapies must overcome challenges such as immune evasion and tumor-mediated immunosuppression [147]. TMEs often suppress T-cell activation and proliferation, undermining therapeutic efficacy [183]. Innovative combination therapies that pair neoantigen-targeting strategies with immune checkpoint inhibitors, such as PD-1/PD-L1 or CTLA-4 blockers, show significant promise in mitigating these barriers [120, 184]. These synergistic approaches enhance immune persistence and activity, yielding improved therapeutic outcomes [185, 186]. Additionally, the use of personalized adjuvants targeting immunosuppressive pathways represents a strategic advancement. These adjuvants, when combined with neoantigen vaccines or adoptive T-cell therapies, amplify immune recognition and response, fostering a more effective anti-tumor immunity [134, 173, 187].

Emerging hybrid strategies that integrate multiple therapeutic modalities are gaining traction, addressing the inherent limitations of individual approaches. For example, combining CAR-T cell therapy with bispecific antibody treatments can overcome the antigen breadth constraints of standalone methods, providing broader tumor coverage and enhancing immune activation [188, 189]. Oncolytic viruses deliver dual benefits by directly targeting tumors and augmenting neoantigen presentation through tumor cell death and systemic immune stimulation [126]. Advances in stealth viral vectors and genetically modified oncolytic viruses address pre-existing immunity challenges, ensuring sustained therapeutic efficacy.

The scalability and accessibility of neoantigen-based therapies are critical for their global adoption. Automated, large-scale manufacturing processes are being developed to reduce production costs and streamline therapy distribution [131, 190]. These efforts aim to democratize access to advanced cancer treatments, ensuring that the benefits of precision immunotherapy reach diverse populations worldwide.

The integration of cutting-edge technologies, multi-omic approaches, and scalable production systems is driving the evolution of neoantigen-based cancer therapies [13, 191, 192]. By addressing existing challenges and harnessing emerging opportunities, the field is on the cusp of delivering highly personalized, effective, and accessible cancer treatments. These advancements mark a paradigm shift in cancer immunotherapy, offering renewed hope for improved patient outcomes across a broad spectrum of malignancies.

## Advanced methodologies and tools for neoantigen prediction in personalized cancer immunotherapy

### The role of neoantigen prediction in personalized cancer immunotherapy

**Figure 4:**
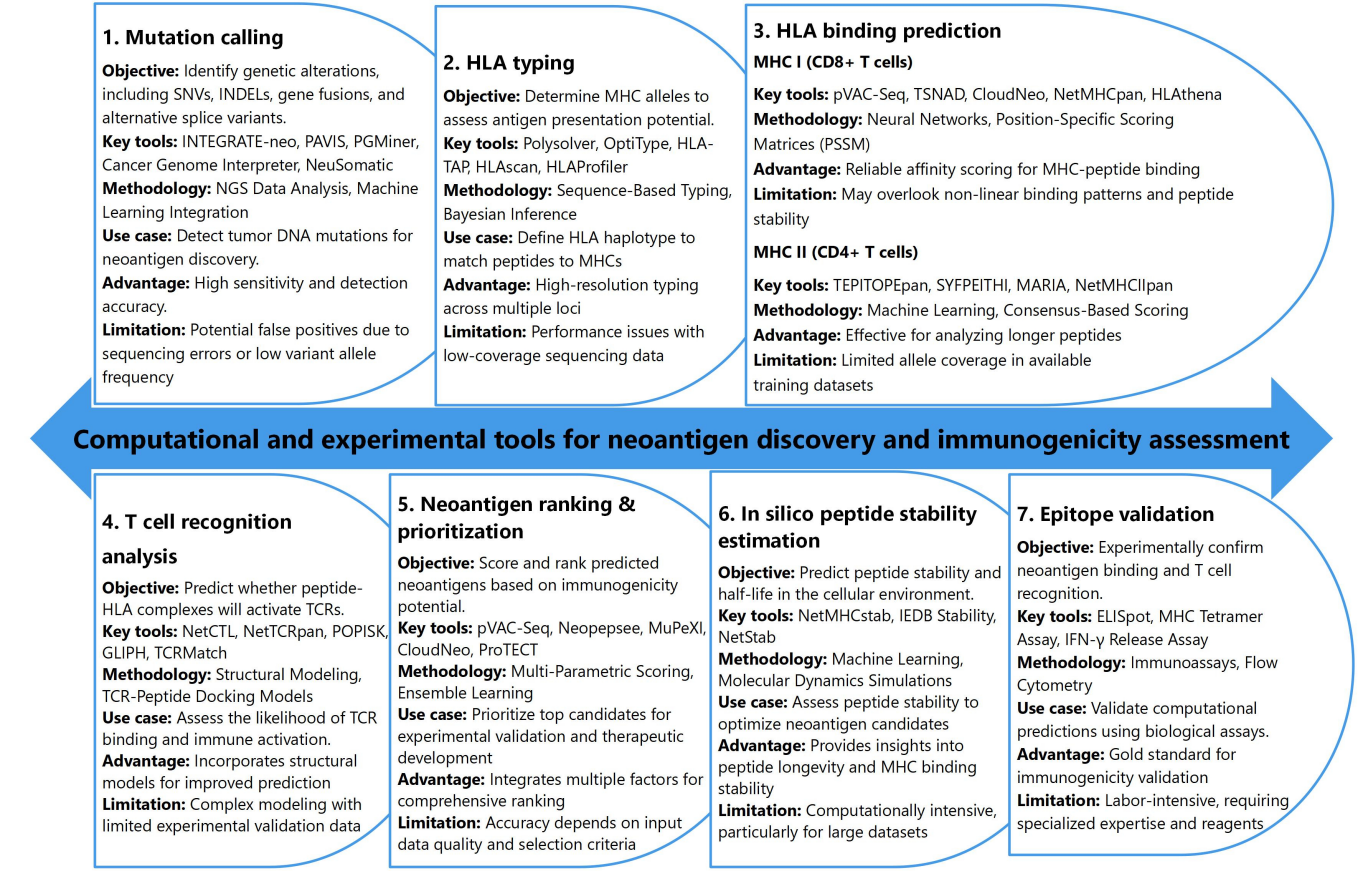
**Overview of computational and experimental tools for neoantigen discovery and immunogenicity assessment.** HLA: human leukocyte antigen; TCR: T-cell receptor; MHC: major histocompatibility complex

In personalized cancer immunotherapy, the identification and targeting of neoantigens are critical for developing tailored therapeutic strategies [13, 193]. The process begins with mutation calling, which involves the detection of genetic alterations, such as SNVs, INDELs, gene fusions, and alternative splice variants, all of which are potential sources of neoantigens [84]. Tools like INTEGRATE-neo, PAVIS, and Cancer Genome Interpreter leverage NGS data and machine learning integration to identify these mutations with high sensitivity [194–196] (Figure 4). However, challenges such as false positives from sequencing errors or low variant allele frequencies can complicate mutation detection, highlighting the need for robust validation methods in the neoantigen discovery pipeline [197].

Once mutations are identified, the next crucial step is human leukocyte antigen (HLA) typing, which determines the MHC alleles expressed by the individual. This is vital for predicting the potential presentation of peptides on the surface of tumor cells [198–200]. High-resolution genotyping tools like Polysolver and OptiType use sequence-based typing and Bayesian inference to accurately determine an individual’s HLA haplotype [201–203] (Figure 4). The accurate matching of peptides to specific MHCs is essential for the subsequent prediction of antigen presentation [17, 204, 205]. However, challenges arise in cases of low-coverage sequencing data, where the performance of HLA typing tools may be limited, necessitating further refinement of these methods for clinical applications.

HLA binding prediction is the next step, focusing on the prediction of peptide-MHC binding affinity and stability. The ability of peptides derived from mutations to bind to MHC molecules plays a pivotal role in eliciting a T-cell-mediated immune response [206, 207]. Tools such as NetMHCpan and pVAC-Seq employ advanced algorithms, including neural networks and position-specific scoring matrices, to predict the binding of peptides to MHC class I molecules (for CD8+ T cells) and class II molecules (for CD4+ T cells) [57, 172, 208] (Figure 4). These tools provide reliable predictions for peptide-MHC affinity, although they may overlook complex, non-linear binding patterns or peptide stability, which could affect the immune response. As a result, the integration of multiple prediction tools and experimental validation becomes necessary for optimizing neoantigen identification and ensuring effective immunogenicity [209].

To further refine neoantigen candidates, T cell recognition analysis assesses the likelihood of TCR binding to peptide-MHC complexes. Tools such as NetCTL and TCRMatch utilize structural modeling and TCR-peptide docking models to predict TCR interaction and immune activation [210, 211]. Despite their usefulness, these models are often complex and face challenges due to limited experimental validation data. Additionally, neoantigen ranking and prioritization tools like Neopepsee and MuPeXI evaluate immunogenicity and expression levels to prioritize the most promising neoantigens for therapeutic development [172, 212, 213]. Finally, the validation of computational predictions through experimental tools like ELISpot and MHC Tetramer assays is essential to confirm the immunogenicity of neoantigens [41, 214, 215]. While these experimental methods provide the gold standard for validation, they are labor-intensive and require specialized expertise and reagents. The integration of computational tools with experimental validation creates a comprehensive approach to neoantigen prediction, ultimately enabling the development of personalized cancer immunotherapies that target specific tumor mutations.

### Computational tools for neoantigen identification and immunogenicity prediction in cancer immunotherapy

In the rapidly advancing field of immunogenomics, computational tools have become indispensable for neoantigen identification and immunogenicity prediction. These tools assist in pinpointing potential therapeutic targets, especially in the context of cancer immunotherapy, by predicting the ability of tumor mutations to be presented on MHC molecules and recognized by the immune system [173, 216]. Table 3 compares several prominent computational tools in this domain, each with specific functions, strengths, and limitations. These tools vary in their approach, from mutation calling and HLA typing to peptide binding prediction and T-cell recognition [217]. Despite their distinct methodologies, all these tools aim to provide accurate predictions that can aid in the development of personalized vaccines or other immunotherapies.

**Table 3:** Comparison of computational tools for neoantigen identification and immunogenicity prediction

**Tool**	**Step**	**Data input**	**Algorithm type**	**Key strength**	**Limitations**	**References**
INTEGRATE-neo	Mutation calling	Whole exome/Genome sequencing	Graph-based detection	High sensitivity for detecting insertions and complex mutations	Limited to specific mutation types; may miss small variants	[218–220]
Polysolver	HLA typing	Whole exome sequencing	Bayesian inference	Highly accurate even with low-coverage data	Computationally intensive, requires high processing power	[201–203]
NetMHCpan	HLA binding prediction	Peptide sequences	Neural network	Broad coverage of MHC alleles across diverse populations	Sequence length constraints may not predict all peptides	[221–224]
NetMHCIIpan	HLA binding prediction	Peptide sequences	Neural network	High sensitivity for MHC class II binding predictions	Limited coverage of MHC class II alleles, not exhaustive	[225, 226]
NetCTL	T cell recognition	Peptide-HLA binding data	Machine learning	Specific scoring for CD8+ T cell recognition	Does not model TCR structure or interactions well	[227]
MHCflurry	HLA binding prediction	Peptide sequences	Machine learning/Deep learning	Accurate binding affinity predictions for class I peptides	Limited prediction capability for rare MHC alleles	[228, 229]
VAXign	Epitope validation	Peptide sequences	Statistical modeling	High throughput capability for large peptide datasets	Requires large amounts of experimental validation data	[230–232]
Immune Epitope Database (IEDB)	Epitope validation	Peptide sequences, HLA typing	Database query	Extensive experimental data support, widely recognized database	Limited by available datasets, may lack specificity in certain cases	[233–235]
NeoepitopePred	T cell recognition	Peptide-HLA binding data	Hybrid model (SVM, neural networks)	Comprehensive T-cell response prediction model	Requires large training datasets, may overestimate some responses	[172, 236, 237]
MHC-I Binding Prediction Tool	HLA binding prediction	Peptide sequences	Weighted scoring system	Effective for a wide range of peptide sequences	Limited by scoring system, may not capture all binding interactions	[238–240]

HLA: human leukocyte antigen; TCR: T-cell receptor; SVM: support vector machines; MHC: major histocompatibility complex

One of the most prominent tools is INTEGRATE-neo, which focuses on mutation calling through whole exome/genome sequencing. It employs graph-based detection, offering high sensitivity for detecting complex mutations and insertions [219]. However, its major limitation is that it is confined to specific types of mutation and may miss smaller variants. Similarly, Polysolver is a robust tool for HLA typing using Bayesian inference, which is known for its high accuracy, even with low-coverage data [201–203]. Despite its effectiveness, it is computationally intensive and demands significant processing power.

Tools such as NetMHCpan and NetMHCIIpan, which focus on HLA binding prediction, rely on neural network algorithms to predict peptide binding across various MHC alleles. While NetMHCpan offers broad allele coverage, it is constrained by sequence length limitations and may fail to predict certain peptides [221, 222]. Meanwhile, NetMHCIIpan is highly sensitive for MHC class II binding predictions, but its coverage is limited, particularly for rare alleles [241–243].

In addition to mutation calling and peptide binding prediction, several tools aim to predict T-cell recognition. NetCTL, based on machine learning, provides specific scoring for CD8+ T-cell recognition; however, it does not account for TCR structure or interactions, which can be a critical factor in immune response [41, 221, 244]. Similarly, NeoepitopePred integrates a hybrid model using support vector machines (SVM) and neural networks to predict T-cell responses, but it requires large training datasets and may overestimate some immune responses [236, 237, 245]. VAXign, a statistical modeling tool for epitope validation, can handle large peptide datasets efficiently but necessitates substantial experimental validation [230–232]. Meanwhile, the Immune Epitope Database (IEDB) is a widely recognized and extensively supported database query tool for epitope validation, although its performance may be hindered by the availability and specificity of datasets [233–235].

Together, these tools provide complementary insights into the complex process of neoantigen identification, each offering unique advantages but also facing significant challenges in terms of data coverage, computational power, and the need for experimental validation.

## Comparative analysis of current therapies for GBM and personalized neoantigen vaccines

GBM is a highly aggressive and treatment-resistant brain cancer, presenting significant challenges in clinical management [246, 247]. Current and emerging therapeutic strategies for GBM encompass a broad spectrum of approaches, each characterized by distinct mechanisms of action, varying levels of efficacy, inherent limitations, and impacts on patient outcomes and quality of life [20, 248, 249]. A thorough evaluation of these therapies highlights the complexity of GBM treatment and underscores the urgent need for innovative and multimodal strategies to achieve meaningful improvements in patient survival and overall well-being.

**Figure 5:**
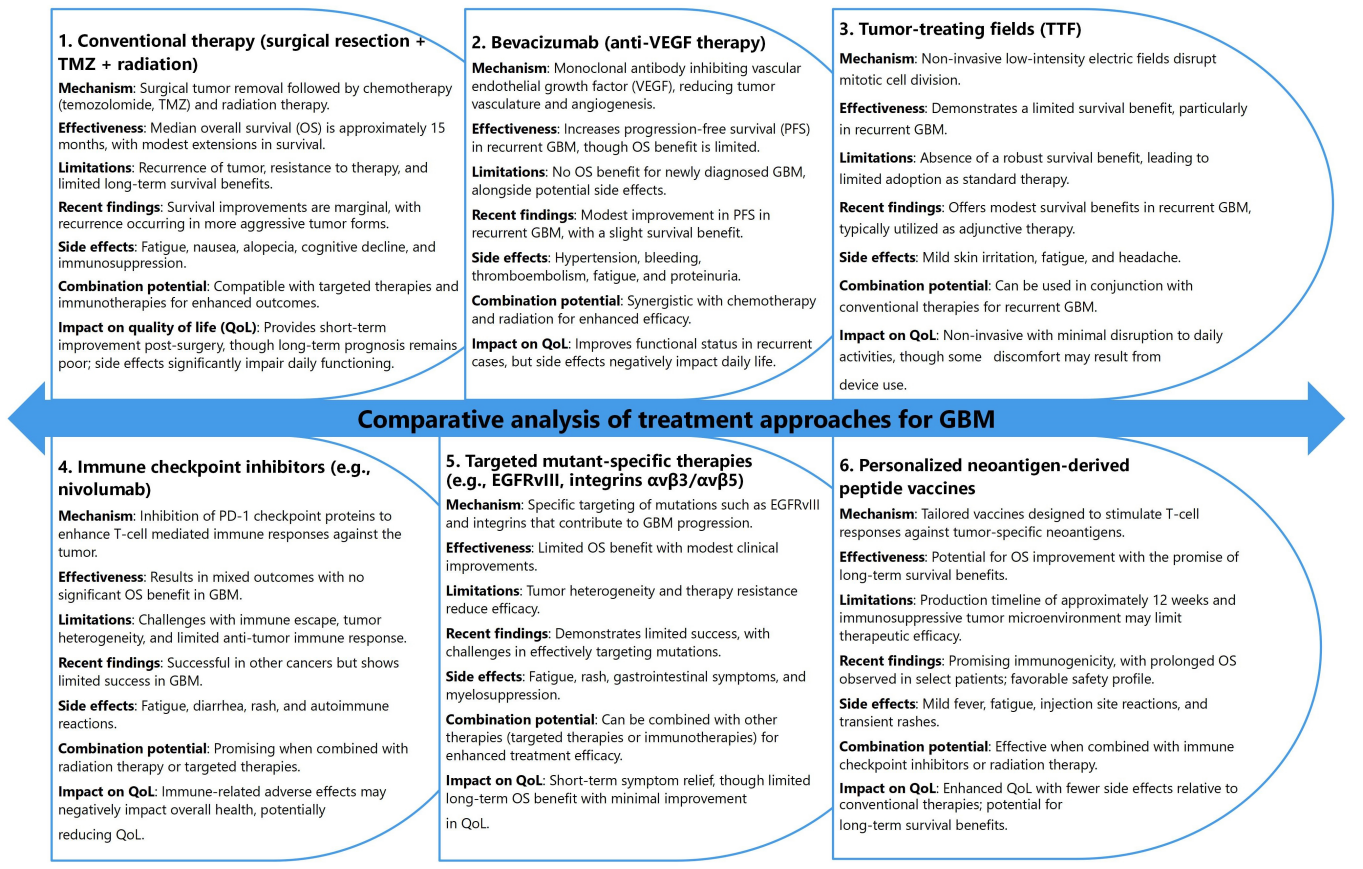
Comparative analysis of treatment approaches for glioblastoma (GBM)

Conventional treatment options, including surgical resection, temozolomide chemotherapy, and radiation therapy, remain the standard of care for GBM patients. While these modalities collectively offer modest survival benefits, with a median overall survival of approximately 15 months, their limitations are pronounced [250]. Tumor heterogeneity and the emergence of therapeutic resistance contribute to inevitable disease recurrence (Figure 5). Moreover, adverse effects, such as cognitive decline, fatigue, and immunosuppression, significantly compromise the quality of life. These challenges underscore the importance of integrating advanced therapeutic approaches to enhance efficacy and address the long-term limitations of these foundational treatments [251, 252].

Emerging therapies, such as bevacizumab, TTF, immune checkpoint inhibitors, and targeted therapies, provide novel avenues for GBM management (Figure 5) [253, 254]. Bevacizumab, an anti-angiogenic monoclonal antibody, has demonstrated promise in improving progression-free survival (PFS) in recurrent GBM cases but has not shown a significant extension of overall survival, particularly in newly diagnosed patients [255, 256]. Similarly, TTF, a non-invasive therapeutic modality that disrupts mitotic processes, achieves only limited survival benefits [257]. Immune checkpoint inhibitors and targeted therapies face significant challenges, including tumor immune evasion and heterogeneity, which limit their standalone efficacy [258, 259]. However, the potential for combination therapies to overcome these obstacles represents a critical area for further research and optimization.

Personalized neoantigen-derived peptide vaccines represent a promising advancement in precision medicine for GBM (Figure 5) [23, 260]. These vaccines leverage patient-specific tumor antigens to elicit robust T-cell responses, offering the potential for extended survival and improved quality of life [23]. Despite their promise, challenges such as the time-intensive production process and the immunosuppressive TME hinder their broad applicability [41, 103]. When combined with complementary therapies like checkpoint inhibitors, these vaccines demonstrate synergistic potential, paving the way for transformative developments in GBM treatment [261, 262].

By critically analyzing the strengths and limitations of these therapeutic modalities, researchers and clinicians can navigate the evolving landscape of GBM management more effectively and identify innovative strategies to enhance patient outcomes. The next section will explore the role of personalized neoantigen-derived peptide vaccines in greater detail.

## Targeting tumor-specific neoantigens in GBM: innovations in immunotherapy, personalized vaccines, and overcoming immunosuppressive barriers

### Therapeutic strategies targeting neoantigens in GBM

Neoantigens have catalyzed the development of advanced immunotherapeutic strategies for GBM. These strategies encompass a spectrum of approaches aimed at enhancing the immune system’s ability to recognize and destroy tumor cells. Personalized cancer vaccines, for instance, utilize patient-specific neoantigens to activate CD8+ and helper T cells, initiating a robust immune response [141]. However, tumor heterogeneity and immune evasion mechanisms remain significant barriers to efficacy (Table 4). To address these challenges, research has integrated vaccines with checkpoint inhibitors, demonstrating improved immune activation in GBM [263]. Future directions involve expanding vaccine applications to other tumor types and refining neoantigen identification processes to enhance immunogenicity.

**Table 4:** Neoantigen-driven immunotherapeutic strategies for glioblastoma

**Therapeutic strategy**	**Mechanism**	**Application in GBM**	**Key challenges**	**Recent advances**	**Future directions**	**References**
Personalized cancer vaccines	Prime the immune system with patient-specific neoantigens.	Induces tumor-specific immune responses by presenting identified neoantigens, activating both CD8+ T cells and helper T cells.	Limited immune response due to tumor heterogeneity and immune evasion mechanisms (e.g., checkpoint inhibition).	Development of combinatory approaches with checkpoint inhibitors to boost efficacy in GBM.	Expansion to other tumor types beyond GBM and improvement in neoantigen identification for higher immunogenicity.	[134]
Adoptive T cell therapies	Infuse patients with expanded T cells specifically targeting neoantigens.	Increases the quantity of tumor-specific T cells, improving tumor cell recognition and destruction through cytotoxic mechanisms.	Difficulty in maintaining T cell persistence and activity within the immunosuppressive GBM tumor microenvironment.	Use of IL-2 or other cytokines to support T cell expansion and persistence in vivo.	Optimizing T cell expansion methods and improving the persistence of T cells within the GBM microenvironment.	[264]
TCR-engineered lymphocytes	Modify T cells ex vivo to express receptors targeting specific neoantigens on tumor cells, then reinfuse them into the patient.	Improves T cell specificity and efficacy in targeting neoantigen-expressing tumor cells, enhancing tumor cell recognition and killing.	Off-target effects and T cell exhaustion in the hostile tumor environment can reduce efficacy.	Advances in TCR optimization to avoid off-target effects and increase the recognition of a broader range of neoantigens in GBM.	Developing next-generation TCR engineering techniques for targeting neoantigens more effectively, along with immune checkpoint blockers.	[149]
Immunotherapy combination therapy	Combine personalized vaccines, adoptive T cells, or TCR-engineered lymphocytes with other immunomodulatory agents (e.g., checkpoint inhibitors).	Provides synergistic effects, improving immune response and overcoming GBM’s immune evasion mechanisms (e.g., PD-1/PD-L1 axis).	Combination therapy may lead to increased toxicity, requiring careful patient monitoring and dosing.	Promising results combining anti-PD-1/PD-L1 with TCR-engineered T cells or vaccines to overcome immune suppression.	Expanding personalized combination therapies to include other immune modulators and identifying the most effective pairings.	[261]
Chimeric antigen receptor (CAR)-T cells	Engineer T cells with a CAR that targets a specific antigen on GBM cells, enhancing their ability to recognize and attack tumors.	Enhances tumor cell recognition and cytotoxicity against neoantigen-expressing GBM cells.	Limited by antigen escape, GBM’s heterogeneous nature, and CAR-T cell exhaustion in the tumor microenvironment.	Development of CAR-T cells targeting novel neoantigens and improving persistence through advanced cytokine support and genetic engineering.	Exploring multi-antigen CAR T cells targeting diverse neoantigens in GBM, along with methods to sustain CAR-T cell activity.	[265]
Oncolytic virus therapy	Use modified viruses that selectively infect and kill tumor cells while stimulating anti-tumor immune responses.	Induces tumor cell death and enhances immune responses to tumor neoantigens, potentially increasing efficacy of vaccines or T cell therapies.	Potential for oncolytic virus resistance and insufficient targeting specificity in the heterogeneous GBM tumor environment.	Oncolytic virus therapies combined with checkpoint inhibitors have shown early promise in preclinical models.	Investigating improved viral vectors that can better target GBM cells and enhance immune activation.	[123]
CRISPR-Cas9 gene editing	Modify the genome of T cells or tumor cells to enhance neoantigen targeting or create new therapeutic pathways for immune evasion.	Potential to enhance the precision and efficiency of T cell therapies or alter GBM’s immune evasion mechanisms to increase treatment efficacy.	Risks of off-target mutations, ethical concerns with gene-editing, and long-term effects of genetic modifications.	Successful use of CRISPR-Cas9 to engineer T cells for better targeting of neoantigens, with promising results in early-phase trials.	Expansion of CRISPR-based approaches to more effectively engineer immune cells and GBM cells for personalized therapies.	[175]
Peptide-based immunotherapies	Use synthetic peptides derived from identified neoantigens to activate immune cells in the tumor microenvironment.	Can specifically activate immune responses targeting neoantigens in GBM, enhancing tumor-specific immunity.	Limited by peptide delivery and the complexity of ensuring effective immune activation against heterogenous tumor populations.	Nanoparticle-mediated peptide delivery systems have enhanced peptide efficacy in preclinical models.	Optimizing delivery methods for peptide-based therapies to ensure efficient and targeted immune activation within GBM tumors.	[52]
Neoantigen-based biomarkers	Utilize identified neoantigens as biomarkers to predict treatment response and monitor therapy efficacy.	Can guide personalized therapy choices, monitor immune response, and track disease progression in GBM patients.	The complexity of tracking neoantigen responses in vivo, and the need for accurate biomarkers to predict treatment outcomes.	Development of liquid biopsy approaches using neoantigen biomarkers for non-invasive tracking of tumor progression and therapy response.	Continued refinement of non-invasive biomarkers for GBM treatment monitoring, including early detection of resistance.	[266]

TCR: T-cell receptor; GBM: glioblastoma

Adoptive T cell therapies and TCR-engineered lymphocytes represent another pivotal class of neoantigen-focused therapies. Adoptive T cell therapies bolster tumor-specific cytotoxicity by infusing patients with expanded T cells targeting GBM-specific neoantigens (Table 4) [149]. However, sustaining T cell activity in GBM’s immunosuppressive environment is challenging [267]. Advances in cytokine supplementation, such as interleukin-2 (IL-2), have shown promise in enhancing T cell persistence and efficacy [268, 269]. Similarly, TCR-engineered lymphocytes improve tumor targeting by expressing receptors specific to neoantigens. Innovations in TCR optimization have minimized off-target effects and expanded the range of neoantigens targeted in GBM [150]. Emerging techniques aim to refine TCR engineering, leveraging combination therapies to further enhance treatment efficacy [270, 271].

CAR-T cells and oncolytic virus therapies highlight the potential of engineered and biologically driven approaches to target neoantigens (Table 4) [272]. CAR-T cells are equipped with receptors that specifically recognize GBM antigens, significantly enhancing tumor cell recognition and destruction [273, 274]. However, antigen escape and tumor heterogeneity present significant obstacles. Advanced CAR designs targeting multiple neoantigens and incorporating genetic modifications to sustain T cell activity are under investigation [275, 276]. Oncolytic virus therapies, meanwhile, employ modified viruses to selectively kill tumor cells and stimulate immune responses [123, 165]. Recent advancements in viral engineering and combinatory use with checkpoint inhibitors have demonstrated enhanced efficacy, particularly in preclinical models, underscoring the potential of synergistic therapeutic combinations [277, 278].

Emerging technologies such as CRISPR-Cas9 gene editing and peptide-based immunotherapies provide further avenues for innovation in neoantigen-targeted strategies [175, 279]. CRISPR-Cas9 has enabled precise modifications in T cells, enhancing neoantigen recognition and therapeutic outcomes in early-phase studies (Table 4) [175, 280]. Future research aims to expand its applications while addressing concerns about off-target effects and long-term safety. Peptide-based immunotherapies utilize synthetic peptides to activate immune cells against GBM-specific neoantigens. However, effective delivery remains a challenge [281, 282]. Nanoparticle-mediated delivery systems have improved peptide stability and target preclinical models, paving the way for clinical translation. Additionally, neoantigen-based biomarkers emerge as tools for monitoring therapy efficacy and guiding personalized treatments (Table 4) [134]. Non-invasive liquid biopsy techniques show potential for tracking tumor progression and detecting resistance, signaling a paradigm shift in GBM management [283].

### Challenges and prospects in advancing neoantigen-based immunotherapies for GBM

Neoantigen-based immunotherapies represent a promising frontier in the treatment of GBM, but their clinical application faces several significant challenges [131, 261]. Among the most formidable obstacles is tumor heterogeneity, a hallmark of GBM that manifests as profound genetic and phenotypic diversity [284, 285]. This heterogeneity enables tumors to adapt swiftly to therapeutic pressures, often leading to immune escape [286]. For instance, tumor cells may lose the expression of targeted neoantigens or develop resistance mechanisms that diminish immune surveillance, ultimately limiting the efficacy of neoantigen-targeted treatments [11, 133]. Such adaptability underscores the need for strategies that can address the dynamic and heterogeneous nature of GBM [287].

The immunosuppressive TME further complicates the application of neoantigen-based immunotherapies [11, 149]. Regulatory T cells (Tregs), myeloid-derived suppressor cells (MDSCs), and immunosuppressive cytokines like TGF-β collectively create a microenvironment that inhibits immune cell activity [288, 289]. This suppression prevents immune cells from fully engaging with and eliminating tumor cells, thereby significantly reducing the effectiveness of therapeutic interventions [290, 291]. In addition, immune evasion mechanisms, such as those mediated by the PD-1/PD-L1 and CTLA-4 pathways, further enable tumors to escape immune detection and suppression [292, 293], presenting yet another barrier to successful treatment.

To overcome these challenges, researchers are exploring combination therapies designed to enhance the efficacy of neoantigen-based immunotherapies [294]. One promising approach involves the use of immune checkpoint inhibitors, such as anti-PD-1 or anti-CTLA-4 antibodies, which block tumor-mediated immune suppression [120, 295]. These inhibitors enhance the immune system’s ability to recognize and target neoantigen-expressing cells, thereby improving therapeutic outcomes [11, 296]. Concurrently, efforts to reprogram the immunosuppressive TME by targeting Tregs, MDSCs, and immunosuppressive cytokines aim to create a more supportive environment for immune-mediated tumor destruction [288, 297].

Personalized neoantigen vaccines have emerged as a particularly compelling therapeutic strategy. These vaccines, which focus on tumor-specific neoantigens, have demonstrated the ability to elicit robust T cell responses in GBM patients, providing evidence of their potential to stimulate tailored immune responses while sparing healthy tissues [131, 161, 261]. However, several critical aspects must be optimized to fully realize their therapeutic potential, including the selection of immunogenic neoantigens, the development of efficient vaccine delivery platforms, and the refinement of dosing strategies [173]. Ensuring that the selected neoantigens are both specific to the patient’s tumor and capable of eliciting strong immune responses is paramount for the success of these therapies.

TCR-engineered lymphocytes offer another promising avenue for advancing neoantigen-based immunotherapy. These modified T cells are engineered to exhibit enhanced specificity and potency, allowing them to selectively target and eliminate neoantigen-expressing GBM cells [149]. To further enhance their effectiveness, researchers are investigating the combination of TCR-engineered lymphocytes with immune checkpoint inhibitors [270, 298]. By blocking tumor-induced immune suppression, these combinations may sustain immune responses and reduce the likelihood of tumor immune escape [299, 300].

Clinical trials investigating neoantigen-targeted therapies in GBM have provided encouraging, though preliminary, results [260, 261]. Personalized neoantigen vaccines have demonstrated their ability to activate immune responses, offering valuable insights into their therapeutic potential [134, 136, 301]. However, long-term clinical outcomes, such as survival benefits, remain to be thoroughly evaluated. Early successes in integrating neoantigen vaccines with other immunotherapeutic approaches highlight the potential of combination strategies, but additional research is needed to refine these approaches and address the immunosuppressive elements of the TME [103, 302]. Overcoming the inhibitory effects of Tregs, MDSCs, and immunosuppressive cytokines like TGF-β will be critical for improving the efficacy of neoantigen-based therapies [303, 304].

By addressing these challenges and leveraging innovative combination strategies, neoantigen-based immunotherapies have the potential to transform GBM treatment and improve patient outcomes. Continued research and clinical investigation will be essential to unlock the full therapeutic promise of these approaches.

## Clinical strategies in neoantigen-based immunotherapies for cancer treatment

Neoantigen-based immunotherapies have emerged as a promising approach for personalized cancer treatment, leveraging TSAs to provoke robust immune responses. Currently, a variety of therapeutic strategies—such as cancer vaccines, ACT, CAR-T therapy, and immune checkpoint blockade (ICB)—are undergoing clinical evaluation (Table 5). These therapies are being assessed for their ability to enhance immune responses, reduce tumor burden, and improve patient outcomes. Clinical trials are closely monitoring the safety, efficacy, and overall clinical impact of these therapies across various cancer types, highlighting the ongoing evolution of neoantigen-targeted immunotherapies (Table 5).

**Table 5:** Clinical trials investigating neoantigen-based immunotherapies

**Therapy type**	**Cancer type(s)**	**Trial identifier(s)**	**Therapy combinations**	**Key outcomes/endpoints**
**Neoantigen-directed cancer vaccines**				
Dendritic cell (DC) vaccine	Melanoma	NCT00683670		Induction of neoantigen-specific T cells
Synthetic long peptide (SLP) vaccine	Melanoma	NCT01970358, NCT02035956		No evidence of disease (NED) in some patients, recurrence in others; response to PD-1 therapy
SLP vaccine (GAPVAC)	Glioblastoma	NCT02149225, NCT02287428		Increased neoantigen-specific T cell response
Ribonucleic acid (RNA) vaccine (IVAC MUTANOME)	Melanoma	NCT02035956		Induction of neoantigen-specific T cells
SLP vaccine (NEO-PV-01)	NSCLC	NCT03380871	Anti-PD-1 & Chemotherapy	Induction of neoantigen-specific T cells
Adenoviral & self-amplifying messenger RNA (samRNA) vectors	Microsatellite-Stable colorectal cancer (MSS-CRC), gastroesophageal adenocarcinoma (GEAC), NSCLC	NCT03639714	Anti-PD-1	One complete response (CR) (GEAC); neoantigen-specific T cell induction
**Neoantigen-directed adoptive cell transfer (ACT)**				
Tumor-infiltrating lymphocyte adoptive cell transfer (TIL-ACT)	Cholangiocarcinoma	NCT01174121	Anti-PD-1	Durable response (~9 years) after retreatment with TIL-ACT
	MSS-CRC	NCT01174121		Regression of all metastases
	Breast cancer	NCT01174121		One CR (>5 years), partial response (PR) in others
T cell receptor-engineered adoptive cell transfer (TCR-ACT)	Pancreatic cancer	NCT04146298		PR, metastasis regression at 6 months
**T cell receptor-engineered T cell therapy (TCR-T)**				
TCR-T	Multiple solid tumors	NCT05194735, NCT05105815, NCT04625205, NCT03171220, NCT05020119		Safety, overall response rate (ORR), disease-free survival (DFS) endpoints
	Various cancers	NCT04102436, NCT04596033, NCT02280811, NCT02858310	Chemotherapy	Response rate, adverse events (AEs), dose-limiting toxicities (DLTs)
	Solid tumors	NCT05349890, NCT04520711, NCT03970382	Immune checkpoint blockade (ICB)	Safety, tolerability, DLTs
	Multiple tumors	NCT03412877, NCT04536922	ICB & Chemotherapy	Response rate, treatment effect
	Hepatocellular carcinoma	NCT03199807	Radiotherapy	AEs
**Tumor-infiltrating lymphocytes (TILs)**				
TIL therapy	Solid tumors	NCT05141474		AEs, serious adverse events (SAEs), treatment-limiting toxicity (TLT)
TILs	Gastrointestinal (GI) & pancreatic cancers	NCT04426669, NCT03658785, NCT02959905	Chemotherapy	Maximum tolerated dose (MTD), ORR, AEs
TILs	Melanoma, NSCLC	NCT03997474, NCT04032847	ICB	AEs
TILs	NSCLC, squamous cell carcinoma (SCC), adenosquamous carcinoma	NCT03215810	ICB & chemotherapy	DLTs
**Chimeric antigen receptor t cell therapy (CAR-T)**				
CAR-T	Glioblastoma multiforme	NCT02844062	Chemotherapy	Safety
**Immune checkpoint blockade (ICB) therapy**				
ICB monotherapy	Various cancers	NCT03600155, NCT02553642, NCT03827044, NCT03718767, NCT03925246, NCT03082534, NCT03357757, NCT03813394, NCT02437279, NCT04825990, NCT03130764, NCT03653052, NCT02113657, NCT03040791, NCT04019964, NCT04293419, NCT04262089		Clinical response, safety, DFS, ORR, progression-free survival (PFS)
ICB	Various cancers	NCT04214249, NCT03978624, NCT02990845, NCT02453620, NCT03409198, NCT05456165, NCT05201612, NCT03832621, NCT03186326, NCT04659382, NCT04262687, NCT05141721, NCT04014530, NCT03918499, NCT03655002, NCT03126812, NCT03554317, NCT04336943, NCT04068194, NCT05317000, NCT02883062	Chemotherapy	Clinical response, MTD, AEs, PFS, ORR
ICB	Cutaneous T-cell lymphoma	NCT03385226	Radiotherapy	ORR
ICB	Colorectal cancer, meningioma, rectal cancer	NCT03854799, NCT03604978, NCT04340401	Chemotherapy & radiotherapy	Pathologic response, MTD, ORR, AEs

The clinical trials focus on five primary therapeutic categories: neoantigen-directed cancer vaccines, adoptive cell transfer, TIL therapy, CAR-T therapy, and ICB therapy. Each approach seeks to harness the body’s immune system to more effectively target cancer, either by stimulating immune responses against neoantigens or by genetically engineering immune cells to recognize and eliminate cancer cells (Table 5). For example, vaccines utilizing DCs, synthetic long peptides (SLPs), and RNA aim to prime the immune system to identify and attack tumors expressing neoantigens [305, 306]. ACT and TCR-engineered adoptive cell transfer (TCR-ACT) approaches involve modifying and expanding T cells to enhance their ability to recognize and kill cancer cells [150]. CAR-T therapy involves engineering T cells to express chimeric receptors that specifically target cancer cells [307], while ICB therapy aims to lift immune system inhibitory signals, enabling immune cells to attack tumor cells more effectively [308].

These clinical trials encompass a wide range of cancer types, including melanoma, GBM, NSCLC, and colorectal cancer, among others (Table 5). The studies also explore various combinations of these therapies with other treatments, such as PD-1 inhibitors, chemotherapy, radiotherapy, and ICB, to further enhance therapeutic efficacy. Clinical outcomes, such as overall response rate (ORR), disease-free survival (DFS), and PFS, are being used to evaluate the success of these therapies.

## Discussion

The increasing focus on neoantigen-based cancer immunotherapies—encompassing vaccines, ACTs, and immune checkpoint inhibitors—highlights their transformative potential in revolutionizing cancer treatment [10, 103]. Neoantigens, by being tumor-specific, allow for precise targeting of malignancies while sparing healthy tissues, offering a more personalized alternative to traditional therapies [133, 145, 301]. However, while promising, several obstacles need to be addressed to maximize the efficacy and applicability of these treatments.

One primary challenge lies in the identification of immunogenic neoantigens and their corresponding TCRs [30, 309]. Current methods, such as NGS and immunopeptidomics, have made strides in identifying neoantigens [9]. Despite these advancements, the accuracy of epitope prediction remains suboptimal, necessitating the refinement of computational workflows [36, 41]. The experimental validation of these antigens is resource-intensive and time-consuming, further complicating progress. Emerging technologies, such as T-Scan, which assess T cell-mediated killing, hold promise for scaling these processes, but a more efficient approach to TCR mapping, high-throughput platforms, and enhanced algorithms is vital for advancing personalized therapies [310, 311].

The financial and time-related constraints associated with personalized neoantigen therapies represent another significant hurdle. While these therapies promise tailored treatments, the high costs and lengthy timelines associated with producing custom vaccines and identifying TCRs hinder their widespread implementation. A promising alternative lies in the development of off-the-shelf therapies targeting public neoantigens, which arise from common mutations across different tumor types [84, 312]. Although such therapies could alleviate cost and time challenges, targeting shared neoantigens across diverse patient populations and creating effective vaccines for these antigens remains a major scientific hurdle [131, 147]. Future research should focus on the development of public neoantigen-based vaccines, bispecific antibodies, and TCR-based therapies to overcome these limitations.

Combination therapies, particularly pairing neoantigen-based vaccines with ICIs, are emerging as a potential solution to improve treatment efficacy. These combination strategies, which may also include chemotherapy or radiation to boost neoantigen presentation, have shown promise in overcoming immune evasion in solid tumors, especially epithelial cancers [12, 131, 145, 313]. However, combining therapies introduces its own set of challenges, particularly with respect to balancing immune activation and managing immune-related adverse effects [122, 314, 315]. Further exploration is needed into immune-modulating agents such as interferons and TLR agonists to determine optimal dosing schedules and improve their therapeutic impact.

Despite the challenges, the future of neoantigen-based immunotherapies is promising. Future research must focus on optimizing the screening and production processes to enhance scalability and reduce costs. Advances in bioinformatics, machine learning, and AI could significantly improve the accuracy of neoantigen prediction and streamline the process of identifying optimal therapeutic targets. Moreover, novel delivery systems, such as nanoparticles and tumor-derived extracellular vesicles (EVs), hold great potential in augmenting immune responses and improving overall therapeutic outcomes [316, 317]. As clinical trials continue, the integration of neoantigen-based therapies into personalized treatment regimens for specific patient populations, such as the elderly, could further improve treatment efficacy, considering distinct disease progression patterns in these individuals.

In conclusion, neoantigen-based immunotherapies hold significant promise as a frontier in cancer treatment, yet several challenges must be addressed to fully realize their potential. Key obstacles include the efficient identification of neoantigens, cost reduction, and the scalability of therapies. Overcoming these hurdles will require continued advancements in bioinformatics, high-throughput technologies, and combination therapeutic strategies. While the future of neoantigen-based cancer immunotherapy appears promising, achieving its full impact will require overcoming technical, logistical, and financial barriers.

To unlock the potential of personalized therapies, substantial investments in infrastructure, computational tools, and collaborative efforts across disciplines are essential. Collaboration between industry partners, academic institutions, and regulatory bodies will play a pivotal role in translating research findings into clinical applications. The challenge of ensuring cost-effectiveness remains critical to providing equitable access to these therapies for all patients.

Looking forward, enhancing the precision of neoantigen identification through improved bioinformatics models and high-throughput technologies represents a promising direction. Additionally, exploring the use of combination therapies—integrating immune checkpoint inhibitors with other immune modulators—could offer a more comprehensive approach to overcoming tumor resistance mechanisms. Expanding the focus to investigate common neoantigens across various cancers could also lead to the development of more broadly applicable therapies. However, challenges related to computational prediction, resource constraints, and the complexity of immune responses must be addressed through continuous innovation and rigorous clinical testing. Ultimately, while substantial work remains, the future of neoantigen-based immunotherapy is bright, with the potential to revolutionize cancer treatment, particularly in the realm of personalized medicine.

## Abbreviations

ACTs: adoptive cell therapies
CAR-T: chimeric antigen receptor T cells therapy
CTLs: cytotoxic T lymphocytes
DC: dendritic cell
GBMs: glioblastomas
HLA: human leukocyte antigen
ICB: immune checkpoint blockade
INDELs: insertions and deletions
MDSCs: myeloid-derived suppressor cells
MHC: major histocompatibility complex
NGS: next-generation sequencing
NSCLC: non-small cell lung cancer
ORFs: novel open reading frames
PD-1: programmed death-1
PFS: progression-free survival
SNVs: single nucleotide variants
TCR-T: T-cell receptor-engineered T cells therapy
TIL: tumor-infiltrating lymphocyte
TME: tumor microenvironment
Tregs: regulatory T cells
TSAs: tumor-specific antigens
TTF: tumor-treating fields

## Declarations

### Copyright

© The Author(s) 2025.

## References

[1] Denis MM, Tolley ND, Bunting M, Schwertz H, Jiang H, Lindemann S (2005). Escaping the nuclear confines: signal-dependent pre-mRNA splicing in anucleate platelets. Cell.

[2] Wang Y, Liu J, Huang BO, Xu Y, Li J, Huang L (2015). Mechanism of alternative splicing and its regulation. Biomed Rep.

[3] Peng Q, Zhou Y, Oyang L, Wu N, Tang Y, Su M (2022). Impacts and mechanisms of alternative mRNA splicing in cancer metabolism, immune response, and therapeutics. Mol Ther.

[4] Baralle FE, Giudice J (2017). Alternative splicing as a regulator of development and tissue identity. Nat Rev Mol Cell Biol.

[5] Jiang W, Chen L (2020). Alternative splicing: Human disease and quantitative analysis from high-throughput sequencing. Comput Struct Biotechnol J.

[6] Choi S, Cho N, Kim E, Kim KK (2023). The role of alternative pre-mRNA splicing in cancer progression. Cancer Cell Int.

[7] Choi S, Cho N, Kim KK (2023). The implications of alternative pre-mRNA splicing in cell signal transduction. Exp Mol Med.

[8] Cui L, Zheng Y, Xu R, Lin Y, Zheng J, Lin P (2024). Alternative pre-mRNA splicing in stem cell function and therapeutic potential: A critical review of current evidence. Int J Biol Macromol.

[9] Xie N, Shen G, Gao W, Huang Z, Huang C, Fu L (2023). Neoantigens: promising targets for cancer therapy. Signal Transduct Target Ther.

[10] Guan H, Wu Y, Li LU, Yang Y, Qiu S, Zhao Z (2023). Tumor neoantigens: Novel strategies for application of cancer immunotherapy. Oncol Res.

[11] Yarchoan M, 3rd BAJ, Lutz ER, Laheru DA, Jaffee EM (2017). Targeting neoantigens to augment antitumour immunity. Nat Rev Cancer.

[12] Peng M, Mo Y, Wang Y, Wu P, Zhang Y, Xiong F (2019). Neoantigen vaccine: an emerging tumor immunotherapy. Mol Cancer.

[13] Lang F, Schrörs B, Löwer M, Türeci Ö, Sahin U (2022). Identification of neoantigens for individualized therapeutic cancer vaccines. Nat Rev Drug Discov.

[14] Zhu Y, Qian Y, Li Z, Li Y, Li B (2021). Neoantigen-reactive T cell: An emerging role in adoptive cellular immunotherapy. MedComm (2020).

[15] Mardis ER (2019). The Impact of Next-Generation Sequencing on Cancer Genomics: From Discovery to Clinic. Cold Spring Harb Perspect Med.

[16] Román ÁC, Benítez DA, Díaz-Pizarro A, Del Valle-Del Pino N, Olivera-Gómez M, Cumplido-Laso G (2024). Next generation sequencing technologies to address aberrant mRNA translation in cancer. NAR Cancer.

[17] Nielsen M, Andreatta M, Peters B, Buus S (2020). Immunoinformatics: Predicting Peptide-MHC Binding. Annu Rev Biomed Data Sci.

[18] Attermann AS, Barra C, Reynisson B, Schultz HS, Leurs U, Lamberth K (2021). Improved prediction of HLA antigen presentation hotspots: Applications for immunogenicity risk assessment of therapeutic proteins. Immunology.

[19] Rominiyi O, Vanderlinden A, Clenton SJ, Bridgewater C, Al-Tamimi Y, Collis SJ (2021). Tumour treating fields therapy for glioblastoma: current advances and future directions. Br J Cancer.

[20] Angom RS, Nakka NMR, Bhattacharya S (2023). Advances in Glioblastoma Therapy: An Update on Current Approaches. Brain Sci.

[21] Yu MW, Quail DF (2021). Immunotherapy for Glioblastoma: Current Progress and Challenges. Front Immunol.

[22] Nelson TA, Dietrich J (2023). Investigational treatment strategies in glioblastoma: progress made and barriers to success. Expert Opin Investig Drugs.

[23] Latzer P, Zelba H, Battke F, Reinhardt A, Shao B, Bartsch O (2024). A real-world observation of patients with glioblastoma treated with a personalized peptide vaccine. Nat Commun.

[24] Bernstock JD, Hoffman SE, Kappel AD, Valdes PA, Essayed W, Klinger NV (2022). Immunotherapy approaches for the treatment of diffuse midline gliomas. Oncoimmunology.

[25] van den Bent M, Saratsis AM, Geurts M, Franceschi E (2024). H3 K27M-altered glioma and diffuse intrinsic pontine glioma: Semi-systematic review of treatment landscape and future directions. Neuro Oncol.

[26] Zolkind P, Dunn GP, Lin T, Griffith M, Griffith OL, Uppaluri R (2017). Neoantigens in immunotherapy and personalized vaccines: Implications for head and neck squamous cell carcinoma. Oral Oncol.

[27] Ward JP, Gubin MM, Schreiber RD (2016). The Role of Neoantigens in Naturally Occurring and Therapeutically Induced Immune Responses to Cancer. Adv Immunol.

[28] Castro A, Zanetti M, Carter H (2021). Neoantigen Controversies. Annu Rev Biomed Data Sci.

[29] Roerden M, Castro AB, Cui Y, Harake N, Kim B, Dye J (2024). Neoantigen architectures define immunogenicity and drive immune evasion of tumors with heterogenous neoantigen expression. J Immunother Cancer.

[30] Kim SH, Lee BR, Kim SM, Kim S, Kim MS, Kim J (2024). The identification of effective tumor-suppressing neoantigens using a tumor-reactive TIL TCR-pMHC ternary complex. Exp Mol Med.

[31] Yin Q, Tang J, Zhu X (2019). Next-generation sequencing technologies accelerate advances in T-cell therapy for cancer. Brief Funct Genomics.

[32] Marcus A, Gowen BG, Thompson TW, Iannello A, Ardolino M, Deng W (2014). Recognition of tumors by the innate immune system and natural killer cells. Adv Immunol.

[33] Javier RT, Butel JS (2008). The history of tumor virology. Cancer Res.

[34] Sim MJW, Sun PD (2022). T Cell Recognition of Tumor Neoantigens and Insights Into T Cell Immunotherapy. Front Immunol.

[35] Ashi MO, Mami-Chouaib F, Corgnac S (2022). Mutant and non-mutant neoantigen-based cancer vaccines: recent advances and future promises. Explor Target Antitumor Ther.

[36] Okada M, Shimizu K, Fujii SI (2022). Identification of Neoantigens in Cancer Cells as Targets for Immunotherapy. Int J Mol Sci.

[37] Veatch JR, Jesernig BL, Kargl J, Fitzgibbon M, Lee SM, Baik C (2019). Endogenous CD4^+^ T Cells Recognize Neoantigens in Lung Cancer Patients, Including Recurrent Oncogenic *KRAS* and *ERBB2* (*Her2*) Driver Mutations. Cancer Immunol Res.

[38] Gros A, Parkhurst MR, Tran E, Pasetto A, Robbins PF, Ilyas S (2016). Prospective identification of neoantigen-specific lymphocytes in the peripheral blood of melanoma patients. Nat Med.

[39] Hu Z, Leet DE, Allesøe RL, Oliveira G, Li S, Luoma AM (2021). Personal neoantigen vaccines induce persistent memory T cell responses and epitope spreading in patients with melanoma. Nat Med.

[40] Biswas N, Chakrabarti S, Padul V, Jones LD, Ashili S (2023). Designing neoantigen cancer vaccines, trials, and outcomes. Front Immunol.

[41] De Mattos-Arruda L, Vazquez M, Finotello F, Lepore R, Porta E, Hundal J (2020). Neoantigen prediction and computational perspectives towards clinical benefit: recommendations from the ESMO Precision Medicine Working Group. Ann Oncol.

[42] Ebrahimi N, Akbari M, Ghanaatian M, Roozbahani Moghaddam P, Adelian S, Borjian Boroujeni M (2022). Development of neoantigens: from identification in cancer cells to application in cancer vaccines. Expert Rev Vaccines.

[43] Davis L, Tarduno A, Lu YC (2021). Neoantigen-Reactive T Cells: The Driving Force behind Successful Melanoma Immunotherapy. Cancers (Basel).

[44] Yossef R, Tran E, Deniger DC, Gros A, Pasetto A, Parkhurst MR (2018). Enhanced detection of neoantigen-reactive T cells targeting unique and shared oncogenes for personalized cancer immunotherapy. JCI Insight.

[45] Cafri G, Gartner JJ, Zaks T, Hopson K, Levin N, Paria BC (2020). mRNA vaccine-induced neoantigen-specific T cell immunity in patients with gastrointestinal cancer. J Clin Invest.

[46] Eshkiki ZS, Agah S, Tabaeian SP, Sedaghat M, Dana F, Talebi A (2022). Neoantigens and their clinical applications in human gastrointestinal cancers. World J Surg Oncol.

[47] Mørk SK, Kadivar M, Bol KF, Draghi A, Westergaard MCW, Skadborg SK (2022). Personalized therapy with peptide-based neoantigen vaccine (EVX-01) including a novel adjuvant, CAF^®^09b, in patients with metastatic melanoma. Oncoimmunology.

[48] Ott PA, Hu Z, Keskin DB, Shukla SA, Sun J, Bozym DJ (2017). An immunogenic personal neoantigen vaccine for patients with melanoma. Nature.

[49] Zhu G, Pei L, Xia H, Tang Q, Bi F (2021). Role of oncogenic KRAS in the prognosis, diagnosis and treatment of colorectal cancer. Mol Cancer.

[50] Sahin IH, Saridogan T, Ayasun R, Syed MP, Gorantla V, Malhotra M (2024). Targeting KRAS Oncogene for Patients With Colorectal Cancer: A New Step Toward Precision Medicine. JCO Oncol Pract.

[51] Ott PA, Hu-Lieskovan S, Chmielowski B, Govindan R, Naing A, Bhardwaj N (2020). A Phase Ib Trial of Personalized Neoantigen Therapy Plus Anti-PD-1 in Patients with Advanced Melanoma, Non-small Cell Lung Cancer, or Bladder Cancer. Cell.

[52] Chen X, Yang J, Wang L, Liu B (2020). Personalized neoantigen vaccination with synthetic long peptides: recent advances and future perspectives. Theranostics.

[53] Boisguérin V, Castle JC, Loewer M, Diekmann J, Mueller F, Britten CM (2014). Translation of genomics-guided RNA-based personalised cancer vaccines: towards the bedside. Br J Cancer.

[54] Roudko V, Greenbaum B, Bhardwaj N (2020). Computational Prediction and Validation of Tumor-Associated Neoantigens. Front Immunol.

[55] Mardis ER (2019). Neoantigens and genome instability: impact on immunogenomic phenotypes and immunotherapy response. Genome Med.

[56] Diederichs S, Bartsch L, Berkmann JC, Fröse K, Heitmann J, Hoppe C (2016). The dark matter of the cancer genome: aberrations in regulatory elements, untranslated regions, splice sites, non-coding RNA and synonymous mutations. EMBO Mol Med.

[57] Richters MM, Xia H, Campbell KM, Gillanders WE, Griffith OL, Griffith M (2019). Best practices for bioinformatic characterization of neoantigens for clinical utility. Genome Med.

[58] Lee M Jr, Ahmad SF, Xu J (2024). Regulation and function of transposable elements in cancer genomes. Cell Mol Life Sci.

[59] Kassiotis G (2014). Endogenous retroviruses and the development of cancer. J Immunol.

[60] Dadush A, Merdler-Rabinowicz R, Gorelik D, Feiglin A, Buchumenski I, Pal LR (2024). DNA and RNA base editors can correct the majority of pathogenic single nucleotide variants. NPJ Genom Med.

[61] Lehner K, Mudrak SV, Minesinger BK, Jinks-Robertson S (2012). Frameshift mutagenesis: the roles of primer-template misalignment and the nonhomologous end-joining pathway in Saccharomyces cerevisiae. Genetics.

[62] Roudko V, Bozkus CC, Orfanelli T, McClain CB, Carr C, O'Donnell T (2020). Shared Immunogenic Poly-Epitope Frameshift Mutations in Microsatellite Unstable Tumors. Cell.

[63] Mistretta B, Rankothgedera S, Castillo M, Rao M, Holloway K, Bhardwaj A (2023). Chimeric RNAs reveal putative neoantigen peptides for developing tumor vaccines for breast cancer. Front Immunol.

[64] Liu S, Heumüller SE, Hossinger A, Müller SA, Buravlova O, Lichtenthaler SF (2023). Reactivated endogenous retroviruses promote protein aggregate spreading. Nat Commun.

[65] Russ E, Iordanskiy S (2023). Endogenous Retroviruses as Modulators of Innate Immunity. Pathogens.

[66] Sciarrillo R, Wojtuszkiewicz A, Assaraf YG, Jansen G, Kaspers GJL, Giovannetti E (2020). The role of alternative splicing in cancer: From oncogenesis to drug resistance. Drug Resist Updat.

[67] Dvinge H, Bradley RK (2015). Widespread intron retention diversifies most cancer transcriptomes. Genome Med.

[68] Lee Y, Gamazon ER, Rebman E, Lee Y, Lee S, Dolan ME (2012). Variants affecting exon skipping contribute to complex traits. PLoS Genet.

[69] Blazquez L, Emmett W, Faraway R, Pineda JMB, Bajew S, Gohr A (2018). Exon Junction Complex Shapes the Transcriptome by Repressing Recursive Splicing. Mol Cell.

[70] Iida N, Okada A, Kobayashi Y, Chiba K, Yatabe Y, Shiraishi Y (2025). Systematically developing a registry of splice-site creating variants utilizing massive publicly available transcriptome sequence data. Nat Commun.

[71] Hujová P, Souček P, Radová L, Kramárek M, Kováčová T, Freiberger T (2021). Nucleotides in both donor and acceptor splice sites are responsible for choice in NAGNAG tandem splice sites. Cell Mol Life Sci.

[72] Zheng JT, Lin CX, Fang ZY, Li HD (2020). Intron Retention as a Mode for RNA-Seq Data Analysis. Front Genet.

[73] Liu Z, Rabadan R (2021). Computing the Role of Alternative Splicing in Cancer. Trends Cancer.

[74] Mathur M, Kim CM, Munro SA, Rudina SS, Sawyer EM, Smolke CD (2019). Programmable mutually exclusive alternative splicing for generating RNA and protein diversity. Nat Commun.

[75] Park E, Pan Z, Zhang Z, Lin L, Xing Y (2018). The Expanding Landscape of Alternative Splicing Variation in Human Populations. Am J Hum Genet.

[76] Shamnas V M, Singh A, Kumar A, Mishra GP, Sinha SK (2024). Exitrons: offering new roles to retained introns-the novel regulators of protein diversity and utility. AoB Plants.

[77] Srivastava R, Budak G, Dash S, Lachke SA, Janga SC (2017). Transcriptome analysis of developing lens reveals abundance of novel transcripts and extensive splicing alterations. Sci Rep.

[78] Gabay O, Shoshan Y, Kopel E, Ben-Zvi U, Mann TD, Bressler N (2022). Landscape of adenosine-to-inosine RNA recoding across human tissues. Nat Commun.

[79] Cheng H, Yu J, Wong CC (2024). Adenosine-to-Inosine RNA editing in cancer: molecular mechanisms and downstream targets. Protein Cell.

[80] Othoum G, Maher CA (2023). CrypticProteinDB: an integrated database of proteome and immunopeptidome derived non-canonical cancer proteins. NAR Cancer.

[81] Yang H, Li Q, Stroup EK, Wang S, Ji Z (2024). Widespread stable noncanonical peptides identified by integrated analyses of ribosome profiling and ORF features. Nat Commun.

[82] Barczak W, Carr SM, Liu G, Munro S, Nicastri A, Lee LN (2023). Long non-coding RNA-derived peptides are immunogenic and drive a potent anti-tumour response. Nat Commun.

[83] Chen Y, Long W, Yang L, Zhao Y, Wu X, Li M (2021). Functional Peptides Encoded by Long Non-Coding RNAs in Gastrointestinal Cancer. Front Oncol.

[84] Aparicio B, Theunissen P, Hervas-Stubbs S, Fortes P, Sarobe P (2024). Relevance of mutation-derived neoantigens and non-classical antigens for anticancer therapies. Hum Vaccin Immunother.

[85] Li YR, Liu MJ (2020). Prevalence of alternative AUG and non-AUG translation initiators and their regulatory effects across plants. Genome Res.

[86] Wright BW, Yi Z, Weissman JS, Chen J (2022). The dark proteome: translation from noncanonical open reading frames. Trends Cell Biol.

[87] Kumar A, Narayanan V, Sekhar A (2020). Characterizing Post-Translational Modifications and Their Effects on Protein Conformation Using NMR Spectroscopy. Biochemistry.

[88] Dunphy K, Dowling P, Bazou D, O'Gorman P (2021). Current Methods of Post-Translational Modification Analysis and Their Applications in Blood Cancers. Cancers (Basel).

[89] Wang TY, Liu Q, Ren Y, Alam SK, Wang L, Zhu Z (2021). A pan-cancer transcriptome analysis of exitron splicing identifies novel cancer driver genes and neoepitopes. Mol Cell.

[90] Öther-Gee Pohl S, Myant KB (2022). Alternative RNA splicing in tumour heterogeneity, plasticity and therapy. Dis Model Mech.

[91] Lang F, Sorn P, Suchan M, Henrich A, Albrecht C, Köhl N (2024). Prediction of tumor-specific splicing from somatic mutations as a source of neoantigen candidates. Bioinform Adv.

[92] Park J, Chung YJ (2019). Identification of neoantigens derived from alternative splicing and RNA modification. Genomics Inform.

[93] Zhang Y, Li L, Mendoza JJ, Wang D, Yan Q, Shi L (2024). Advances in A-to-I RNA editing in cancer. Mol Cancer.

[94] Cai Y, Li D, Lv D, Yu J, Ma Y, Jiang T (2024). MHC-I-presented non-canonical antigens expand the cancer immunotherapy targets in acute myeloid leukemia. Sci Data.

[95] Ouspenskaia T, Law T, Clauser KR, Klaeger S, Sarkizova S, Aguet F (2022). Unannotated proteins expand the MHC-I-restricted immunopeptidome in cancer. Nat Biotechnol.

[96] Minati R, Perreault C, Thibault P (2020). A Roadmap Toward the Definition of Actionable Tumor-Specific Antigens. Front Immunol.

[97] Lee TA, Tsai EY, Liu SH, Hsu Hung SD, Chang SJ, Chao CH (2024). Post-translational Modification of PD-1: Potential Targets for Cancer Immunotherapy. Cancer Res.

[98] Srivastava AK, Guadagnin G, Cappello P, Novelli F (2022). Post-Translational Modifications in Tumor-Associated Antigens as a Platform for Novel Immuno-Oncology Therapies. Cancers (Basel).

[99] Wirth TC, Kühnel F (2017). Neoantigen Targeting-Dawn of a New Era in Cancer Immunotherapy?. Front Immunol.

[100] Fu D, Calvo JA, Samson LD (2012). Balancing repair and tolerance of DNA damage caused by alkylating agents. Nat Rev Cancer.

[101] Peng Y, Pei H (2021). DNA alkylation lesion repair: outcomes and implications in cancer chemotherapy. J Zhejiang Univ Sci B.

[102] Cheung-Ong K, Giaever G, Nislow C (2013). DNA-damaging agents in cancer chemotherapy: serendipity and chemical biology. Chem Biol.

[103] Fan T, Zhang M, Yang J, Zhu Z, Cao W, Dong C (2023). Therapeutic cancer vaccines: advancements, challenges, and prospects. Signal Transduct Target Ther.

[104] O'Donnell T, Christie EL, Ahuja A, Buros J, Aksoy BA, Bowtell DDL (2018). Chemotherapy weakly contributes to predicted neoantigen expression in ovarian cancer. BMC Cancer.

[105] Czajkowski D, Szmyd R, Gee HE (2022). Impact of DNA damage response defects in cancer cells on response to immunotherapy and radiotherapy. J Med Imaging Radiat Oncol.

[106] Donlon NE, Power R, Hayes C, Reynolds JV, Lysaght J (2021). Radiotherapy, immunotherapy, and the tumour microenvironment: Turning an immunosuppressive milieu into a therapeutic opportunity. Cancer Lett.

[107] Chan Wah Hak CML, Rullan A, Patin EC, Pedersen M, Melcher AA, Harrington KJ (2022). Enhancing anti-tumour innate immunity by targeting the DNA damage response and pattern recognition receptors in combination with radiotherapy. Front Oncol.

[108] Jungles KM, Holcomb EA, Pearson AN, Jungles KR, Bishop CR, Pierce LJ (2022). Updates in combined approaches of radiotherapy and immune checkpoint inhibitors for the treatment of breast cancer. Front Oncol.

[109] Nguyen AT, Shiao SL, McArthur HL (2021). Advances in Combining Radiation and Immunotherapy in Breast Cancer. Clin Breast Cancer.

[110] Majeed H, Gupta V (2025). Adverse Effects of Radiation Therapy.

[111] Dalmasso G, Cougnoux A, Delmas J, Darfeuille-Michaud A, Bonnet R (2014). The bacterial genotoxin colibactin promotes colon tumor growth by modifying the tumor microenvironment. Gut Microbes.

[112] Dougherty MW, Valdés-Mas R, Wernke KM, Gharaibeh RZ, Yang Y, Brant JO (2023). The microbial genotoxin colibactin exacerbates mismatch repair mutations in colorectal tumors. Neoplasia.

[113] Ou S, Wang H, Tao Y, Luo K, Ye J, Ran S (2022). Fusobacterium nucleatum and colorectal cancer: From phenomenon to mechanism. Front Cell Infect Microbiol.

[114] Ranjbar M, Salehi R, Haghjooy Javanmard S, Rafiee L, Faraji H, Jafarpor S (2021). The dysbiosis signature of Fusobacterium nucleatum in colorectal cancer-cause or consequences? A systematic review. Cancer Cell Int.

[115] Lozenov S, Krastev B, Nikolaev G, Peshevska-Sekulovska M, Peruhova M, Velikova T (2023). Gut Microbiome Composition and Its Metabolites Are a Key Regulating Factor for Malignant Transformation, Metastasis and Antitumor Immunity. Int J Mol Sci.

[116] Tan HY, Wang N, Lam W, Guo W, Feng Y, Cheng YC (2018). Targeting tumour microenvironment by tyrosine kinase inhibitor. Mol Cancer.

[117] Shyam Sunder S, Sharma UC, Pokharel S (2023). Adverse effects of tyrosine kinase inhibitors in cancer therapy: pathophysiology, mechanisms and clinical management. Signal Transduct Target Ther.

[118] O'Brien S, Berman E, Moore JO, Pinilla-Ibarz J, Radich JP, Shami PJ (2011). NCCN Task Force report: tyrosine kinase inhibitor therapy selection in the management of patients with chronic myelogenous leukemia. J Natl Compr Canc Netw.

[119] Zhou Y, Tao L, Qiu J, Xu J, Yang X, Zhang Y (2024). Tumor biomarkers for diagnosis, prognosis and targeted therapy. Signal Transduct Target Ther.

[120] Wojtukiewicz MZ, Rek MM, Karpowicz K, Górska M, Polityńska B, Wojtukiewicz AM (2021). Inhibitors of immune checkpoints-PD-1, PD-L1, CTLA-4-new opportunities for cancer patients and a new challenge for internists and general practitioners. Cancer Metastasis Rev.

[121] Wei SC, Duffy CR, Allison JP (2018). Fundamental Mechanisms of Immune Checkpoint Blockade Therapy. Cancer Discov.

[122] Yin Q, Wu L, Han L, Zheng X, Tong R, Li L (2023). Immune-related adverse events of immune checkpoint inhibitors: a review. Front Immunol.

[123] Lin D, Shen Y, Liang T (2023). Oncolytic virotherapy: basic principles, recent advances and future directions. Signal Transduct Target Ther.

[124] Chen L, Zuo M, Zhou Q, Wang Y (2023). Oncolytic virotherapy in cancer treatment: challenges and optimization prospects. Front Immunol.

[125] Palanivelu L, Liu CH, Lin LT (2023). Immunogenic cell death: The cornerstone of oncolytic viro-immunotherapy. Front Immunol.

[126] Tian Y, Xie D, Yang L (2022). Engineering strategies to enhance oncolytic viruses in cancer immunotherapy. Signal Transduct Target Ther.

[127] Aspeslagh S, Morel D, Soria J, Postel-Vinay S (2018). Epigenetic modifiers as new immunomodulatory therapies in solid tumours. Ann Oncol.

[128] Llinàs-Arias P, Íñiguez-Muñoz S, McCann K, Voorwerk L, Orozco JIJ, Ensenyat-Mendez M (2021). Epigenetic Regulation of Immunotherapy Response in Triple-Negative Breast Cancer. Cancers (Basel).

[129] Yang J, Xu J, Wang W, Zhang B, Yu X, Shi S (2023). Epigenetic regulation in the tumor microenvironment: molecular mechanisms and therapeutic targets. Signal Transduct Target Ther.

[130] Liu Z, Ren Y, Weng S, Xu H, Li L, Han X (2022). A New Trend in Cancer Treatment: The Combination of Epigenetics and Immunotherapy. Front Immunol.

[131] Li X, You J, Hong L, Liu W, Guo P, Hao X (2023). Neoantigen cancer vaccines: a new star on the horizon. Cancer Biol Med.

[132] Chehelgerdi M, Chehelgerdi M (2023). The use of RNA-based treatments in the field of cancer immunotherapy. Mol Cancer.

[133] Gupta RG, Li F, Roszik J, Lizée G (2021). Exploiting Tumor Neoantigens to Target Cancer Evolution: Current Challenges and Promising Therapeutic Approaches. Cancer Discov.

[134] Blass E, Ott PA (2021). Advances in the development of personalized neoantigen-based therapeutic cancer vaccines. Nat Rev Clin Oncol.

[135] Lin X, Tang S, Guo Y, Tang R, Li Z, Pan X (2024). Personalized neoantigen vaccine enhances the therapeutic efficacy of bevacizumab and anti-PD-1 antibody in advanced non-small cell lung cancer. Cancer Immunol Immunother.

[136] Wu DW, Jia SP, Xing SJ, Ma HL, Wang X, Tang QY (2024). Personalized neoantigen cancer vaccines: current progression, challenges and a bright future. Clin Exp Med.

[137] Aizaz M, Khan AS, Khan M, Musazade E, Yang G (2024). Advancements in tumor-infiltrating lymphocytes: Historical insights, contemporary milestones, and future directions in oncology therapy. Crit Rev Oncol Hematol.

[138] Kazemi MH, Sadri M, Najafi A, Rahimi A, Baghernejadan Z, Khorramdelazad H (2022). Tumor-infiltrating lymphocytes for treatment of solid tumors: It takes two to tango?. Front Immunol.

[139] Zaborowski AM, Winter DC, Lynch L (2021). The therapeutic and prognostic implications of immunobiology in colorectal cancer: a review. Br J Cancer.

[140] Zhao Y, Deng J, Rao S, Guo S, Shen J, Du F (2022). Tumor Infiltrating Lymphocyte (TIL) Therapy for Solid Tumor Treatment: Progressions and Challenges. Cancers (Basel).

[141] Hu Z, Ott PA, Wu CJ (2018). Towards personalized, tumour-specific, therapeutic vaccines for cancer. Nat Rev Immunol.

[142] Yu C, Liu X, Yang J, Zhang M, Jin H, Ma X (2019). Combination of Immunotherapy With Targeted Therapy: Theory and Practice in Metastatic Melanoma. Front Immunol.

[143] Wu Y, Yu G, Jin K, Qian J (2024). Advancing non-small cell lung cancer treatment: the power of combination immunotherapies. Front Immunol.

[144] Whiteside TL, Demaria S, Rodriguez-Ruiz ME, Zarour HM, Melero I (2016). Emerging Opportunities and Challenges in Cancer Immunotherapy. Clin Cancer Res.

[145] Zhao X, Pan X, Wang Y, Zhang Y (2021). Targeting neoantigens for cancer immunotherapy. Biomark Res.

[146] Zhang Z, Lu M, Qin Y, Gao W, Tao L, Su W (2021). Neoantigen: A New Breakthrough in Tumor Immunotherapy. Front Immunol.

[147] Lybaert L, Lefever S, Fant B, Smits E, De Geest B, Breckpot K (2023). Challenges in neoantigen-directed therapeutics. Cancer Cell.

[148] Lussier DM, Alspach E, Ward JP, Miceli AP, Runci D, White JM (2021). Radiation-induced neoantigens broaden the immunotherapeutic window of cancers with low mutational loads. Proc Natl Acad Sci U S A.

[149] Pang Z, Lu MM, Zhang Y, Gao Y, Bai JJ, Gu JY (2023). Neoantigen-targeted TCR-engineered T cell immunotherapy: current advances and challenges. Biomark Res.

[150] Shafer P, Kelly LM, Hoyos V (2022). Cancer Therapy With TCR-Engineered T Cells: Current Strategies, Challenges, and Prospects. Front Immunol.

[151] Korell F, Berger TR, Maus MV (2022). Understanding CAR T cell-tumor interactions: Paving the way for successful clinical outcomes. Med.

[152] Huang Q, Cai WQ, Han ZW, Wang MY, Zhou Y, Cheng JT (2021). Bispecific T cell engagers and their synergistic tumor immunotherapy with oncolytic viruses. Am J Cancer Res.

[153] Huehls AM, Coupet TA, Sentman CL (2015). Bispecific T-cell engagers for cancer immunotherapy. Immunol Cell Biol.

[154] Kamigaki T, Takimoto R, Okada S, Ibe H, Oguma E, Goto S (2024). Personalized Dendritic-cell-based Vaccines Targeting Cancer Neoantigens. Anticancer Res.

[155] Wang S, Sun J, Chen K, Ma P, Lei Q, Xing S (2021). Perspectives of tumor-infiltrating lymphocyte treatment in solid tumors. BMC Med.

[156] Betof Warner A, Corrie PG, Hamid O (2023). Tumor-Infiltrating Lymphocyte Therapy in Melanoma: Facts to the Future. Clin Cancer Res.

[157] Santos PM, Butterfield LH (2018). Dendritic Cell-Based Cancer Vaccines. J Immunol.

[158] Lee KW, Yam JWP, Mao X (2023). Dendritic Cell Vaccines: A Shift from Conventional Approach to New Generations. Cells.

[159] Ingels J, De Cock L, Stevens D, Mayer RL, Théry F, Sanchez GS (2024). Neoantigen-targeted dendritic cell vaccination in lung cancer patients induces long-lived T cells exhibiting the full differentiation spectrum. Cell Rep Med.

[160] Ramirez CA, Becker-Hapak M, Singhal K, Russler-Germain DA, Frenkel F, Barnell EK (2024). Neoantigen landscape supports feasibility of personalized cancer vaccine for follicular lymphoma. Blood Adv.

[161] Fritsch EF, Burkhardt UE, Hacohen N, Wu CJ (2020). Personal Neoantigen Cancer Vaccines: A Road Not Fully Paved. Cancer Immunol Res.

[162] Li J, Zhang Y, Fu T, Xing G, Cai H, Li K (2024). Clinical advances and challenges associated with TCR-T cell therapy for cancer treatment. Front Immunol.

[163] Bui TA, Mei H, Sang R, Ortega DG, Deng W (2024). Advancements and challenges in developing in vivo CAR T cell therapies for cancer treatment. EBioMedicine.

[164] Lan HR, Chen M, Yao SY, Chen JX, Jin KT (2023). Bispecific antibodies revolutionizing breast cancer treatment: a comprehensive overview. Front Immunol.

[165] Kaufman HL, Kohlhapp FJ, Zloza A (2015). Oncolytic viruses: a new class of immunotherapy drugs. Nat Rev Drug Discov.

[166] Marelli G, Howells A, Lemoine NR, Wang Y (2018). Oncolytic Viral Therapy and the Immune System: A Double-Edged Sword Against Cancer. Front Immunol.

[167] Liu D, Liu L, Li X, Wang S, Wu G, Che X (2024). Advancements and Challenges in Peptide-Based Cancer Vaccination: A Multidisciplinary Perspective. Vaccines (Basel).

[168] Chen P, Liu X, Sun Y, Zhou P, Wang Y, Zhang Y (2016). Dendritic cell targeted vaccines: Recent progresses and challenges. Hum Vaccin Immunother.

[169] Richard G, Princiotta MF, Bridon D, Martin WD, Steinberg GD, De Groot AS (2022). Neoantigen-based personalized cancer vaccines: the emergence of precision cancer immunotherapy. Expert Rev Vaccines.

[170] Garg P, Singhal G, Pareek S, Kulkarni P, Horne D, Nath A (2025). Unveiling the potential of gene editing techniques in revolutionizing Cancer treatment: A comprehensive overview. Biochim Biophys Acta Rev Cancer.

[171] Fang X, Guo Z, Liang J, Wen J, Liu Y, Guan X (2022). Neoantigens and their potential applications in tumor immunotherapy. Oncol Lett.

[172] Cai Y, Chen R, Gao S, Li W, Liu Y, Su G (2023). Artificial intelligence applied in neoantigen identification facilitates personalized cancer immunotherapy. Front Oncol.

[173] Kumar A, Dixit S, Srinivasan K, M D, Vincent PMDR (2024). Personalized cancer vaccine design using AI-powered technologies. Front Immunol.

[174] Liao J, Li X, Gan Y, Han S, Rong P, Wang W (2023). Artificial intelligence assists precision medicine in cancer treatment. Front Oncol.

[175] Feng X, Li Z, Liu Y, Chen D, Zhou Z (2024). CRISPR/Cas9 technology for advancements in cancer immunotherapy: from uncovering regulatory mechanisms to therapeutic applications. Exp Hematol Oncol.

[176] Augustin RC, Cai WL, Luke JJ, Bao R (2024). Facts and Hopes in Using Omics to Advance Combined Immunotherapy Strategies. Clin Cancer Res.

[177] Karimi MR, Karimi AH, Abolmaali S, Sadeghi M, Schmitz U (2022). Prospects and challenges of cancer systems medicine: from genes to disease networks. Brief Bioinform.

[178] Huber F, Arnaud M, Stevenson BJ, Michaux J, Benedetti F, Thevenet J (2024). A comprehensive proteogenomic pipeline for neoantigen discovery to advance personalized cancer immunotherapy. Nat Biotechnol.

[179] Terai YL, Huang C, Wang B, Kang X, Han J, Douglass J (2022). Valid-NEO: A Multi-Omics Platform for Neoantigen Detection and Quantification from Limited Clinical Samples. Cancers (Basel).

[180] McGrail DJ, Federico L, Li Y, Dai H, Lu Y, Mills GB (2018). Multi-omics analysis reveals neoantigen-independent immune cell infiltration in copy-number driven cancers. Nat Commun.

[181] Teplensky MH, Evangelopoulos M, Dittmar JW, Forsyth CM, Sinegra AJ, Wang S (2023). Multi-antigen spherical nucleic acid cancer vaccines. Nat Biomed Eng.

[182] Motamedi H, Ari MM, Shahlaei M, Moradi S, Farhadikia P, Alvandi A (2023). Designing multi-epitope vaccine against important colorectal cancer (CRC) associated pathogens based on immunoinformatics approach. BMC Bioinformatics.

[183] Bader JE, Voss K, Rathmell JC (2020). Targeting Metabolism to Improve the Tumor Microenvironment for Cancer Immunotherapy. Mol Cell.

[184] Marshall HT, Djamgoz MBA (2018). Immuno-Oncology: Emerging Targets and Combination Therapies. Front Oncol.

[185] Rotte A (2019). Combination of CTLA-4 and PD-1 blockers for treatment of cancer. J Exp Clin Cancer Res.

[186] Cheng W, Kang K, Zhao A, Wu Y (2024). Dual blockade immunotherapy targeting PD-1/PD-L1 and CTLA-4 in lung cancer. J Hematol Oncol.

[187] Liu Z, Lv J, Dang Q, Liu L, Weng S, Wang L (2022). Engineering neoantigen vaccines to improve cancer personalized immunotherapy. Int J Biol Sci.

[188] Nath K, Mailankody S, Usmani SZ (2023). The Role of Chimeric Antigen Receptor T-Cell Therapy in the Era of Bispecific Antibodies. Hematol Oncol Clin North Am.

[189] Al Hadidi S, Heslop HE, Brenner MK, Suzuki M (2024). Bispecific antibodies and autologous chimeric antigen receptor T cell therapies for treatment of hematological malignancies. Mol Ther.

[190] Ali A, DiPersio JF (2024). ReCARving the future: bridging CAR T-cell therapy gaps with synthetic biology, engineering, and economic insights. Front Immunol.

[191] Rashid MM, Selvarajoo K (2024). Advancing drug-response prediction using multi-modal and -omics machine learning integration (MOMLIN): a case study on breast cancer clinical data. Brief Bioinform.

[192] Li Y, Wu X, Fang D, Luo Y (2024). Informing immunotherapy with multi-omics driven machine learning. NPJ Digit Med.

[193] Chen F, Zou Z, Du J, Su S, Shao J, Meng F (2019). Neoantigen identification strategies enable personalized immunotherapy in refractory solid tumors. J Clin Invest.

[194] Park Y, Heider D, Hauschild AC (2021). Integrative Analysis of Next-Generation Sequencing for Next-Generation Cancer Research toward Artificial Intelligence. Cancers (Basel).

[195] Clark AJ, Lillard JW Jr (2024). A Comprehensive Review of Bioinformatics Tools for Genomic Biomarker Discovery Driving Precision Oncology. Genes (Basel).

[196] Huang J, Mao L, Lei Q, Guo AY (2024). Bioinformatics tools and resources for cancer and application. Chin Med J (Engl).

[197] Chen I, Chen MY, Goedegebuure SP, Gillanders WE (2021). Challenges targeting cancer neoantigens in 2021: a systematic literature review. Expert Rev Vaccines.

[198] Macy AM, Herrmann LM, Adams AC, Hastings KT (2023). Major histocompatibility complex class II in the tumor microenvironment: functions of nonprofessional antigen-presenting cells. Curr Opin Immunol.

[199] Story CM, Wang T, Bhatt VR, Battiwalla M, Badawy SM, Kamoun M, Center for International Blood and Marrow Transplant Research Immunobiology Working Committee (2021). Genetics of HLA Peptide Presentation and Impact on Outcomes in HLA-Matched Allogeneic Hematopoietic Cell Transplantation. Transplant Cell Ther.

[200] Karnaukhov V, Paes W, Woodhouse IB, Partridge T, Nicastri A, Brackenridge S (2022). HLA variants have different preferences to present proteins with specific molecular functions which are complemented in frequent haplotypes. Front Immunol.

[201] Wang S, Wang M, Chen L, Pan G, Wang Y, Li SC (2023). SpecHLA enables full-resolution HLA typing from sequencing data. Cell Rep Methods.

[202] Matey-Hernandez ML, Danish Pan Genome C, Brunak S, Izarzugaza JMG (2018). Benchmarking the HLA typing performance of Polysolver and Optitype in 50 Danish parental trios. BMC Bioinformatics.

[203] Chen J, Madireddi S, Nagarkar D, Migdal M, Heiden JV, Chang D (2021). In silico tools for accurate HLA and KIR inference from clinical sequencing data empower immunogenetics on individual-patient and population scales. Brief Bioinform.

[204] Racle J, Guillaume P, Schmidt J, Michaux J, Larabi A, Lau K (2023). Machine learning predictions of MHC-II specificities reveal alternative binding mode of class II epitopes. Immunity.

[205] Mikhaylov V, Brambley CA, Keller GLJ, Arbuiso AG, Weiss LI, Baker BM (2024). Accurate modeling of peptide-MHC structures with AlphaFold. Structure.

[206] Mei S, Li F, Leier A, Marquez-Lago TT, Giam K, Croft NP (2020). A comprehensive review and performance evaluation of bioinformatics tools for HLA class I peptide-binding prediction. Brief Bioinform.

[207] Koşaloğlu-Yalçın Z, Lee J, Greenbaum J, Schoenberger SP, Miller A, Kim YJ (2022). Combined assessment of MHC binding and antigen abundance improves T cell epitope predictions. iScience.

[208] Fotakis G, Trajanoski Z, Rieder D (2021). Computational cancer neoantigen prediction: current status and recent advances. Immunooncol Technol.

[209] Hao Q, Wei P, Shu Y, Zhang YG, Xu H, Zhao J (2021). Improvement of Neoantigen Identification Through Convolution Neural Network. Front Immunol.

[210] Bradley P (2023). Structure-based prediction of T cell receptor:peptide-MHC interactions. Elife.

[211] Szeto C, Lobos CA, Nguyen AT, Gras S (2020). TCR Recognition of Peptide-MHC-I: Rule Makers and Breakers. Int J Mol Sci.

[212] BBjerregaard AM, Nielsen M, Hadrup SR, Szallasi Z, Eklund AC (2017). MuPeXI: prediction of neo-epitopes from tumor sequencing data. Cancer Immunol Immunother.

[213] Zhou C, Wei Z, Zhang Z, Zhang B, Zhu C, Chen K (2019). pTuneos: prioritizing tumor neoantigens from next-generation sequencing data. Genome Med.

[214] Smith CC, Chai S, Washington AR, Lee SJ, Landoni E, Field K (2019). Machine-Learning Prediction of Tumor Antigen Immunogenicity in the Selection of Therapeutic Epitopes. Cancer Immunol Res.

[215] Schmidt J, Smith AR, Magnin M, Racle J, Devlin JR, Bobisse S (2021). Prediction of neo-epitope immunogenicity reveals TCR recognition determinants and provides insight into immunoediting. Cell Rep Med.

[216] Shah RK, Cygan E, Kozlik T, Colina A, Zamora AE (2023). Utilizing immunogenomic approaches to prioritize targetable neoantigens for personalized cancer immunotherapy. Front Immunol.

[217] Müller M, Huber F, Arnaud M, Kraemer AI, Altimiras ER, Michaux J (2023). Machine learning methods and harmonized datasets improve immunogenic neoantigen prediction. Immunity.

[218] Karasaki T, Nagayama K, Kuwano H, Nitadori JI, Sato M, Anraku M (2017). Prediction and prioritization of neoantigens: integration of RNA sequencing data with whole-exome sequencing. Cancer Sci.

[219] Zhang J, Mardis ER, Maher CA (2017). INTEGRATE-neo: a pipeline for personalized gene fusion neoantigen discovery. Bioinformatics.

[220] Anzar I, Sverchkova A, Stratford R, Clancy T (2019). NeoMutate: an ensemble machine learning framework for the prediction of somatic mutations in cancer. BMC Med Genomics.

[221] Jurtz V, Paul S, Andreatta M, Marcatili P, Peters B, Nielsen M (2017). NetMHCpan-4.0: Improved Peptide-MHC Class I Interaction Predictions Integrating Eluted Ligand and Peptide Binding Affinity Data. J Immunol.

[222] Hoof I, Peters B, Sidney J, Pedersen LE, Sette A, Lund O (2009). NetMHCpan, a method for MHC class I binding prediction beyond humans. Immunogenetics.

[223] Zeng H, Gifford DK (2019). Quantification of Uncertainty in Peptide-MHC Binding Prediction Improves High-Affinity Peptide Selection for Therapeutic Design. Cell Syst.

[224] Nielsen M, Andreatta M (2016). NetMHCpan-3.0; improved prediction of binding to MHC class I molecules integrating information from multiple receptor and peptide length datasets. Genome Med.

[225] Reynisson B, Alvarez B, Paul S, Peters B, Nielsen M (2020). NetMHCpan-4.1 and NetMHCIIpan-4.0: improved predictions of MHC antigen presentation by concurrent motif deconvolution and integration of MS MHC eluted ligand data. Nucleic Acids Res.

[226] Nielsen M, Justesen S, Lund O, Lundegaard C, Buus S (2010). NetMHCIIpan-2.0 - Improved pan-specific HLA-DR predictions using a novel concurrent alignment and weight optimization training procedure. Immunome Res.

[227] Stranzl T, Larsen MV, Lundegaard C, Nielsen M (2010). NetCTLpan: pan-specific MHC class I pathway epitope predictions. Immunogenetics.

[228] O’Donnell TJ, Rubinsteyn A, Laserson U (2020). MHCflurry 2.0: Improved Pan-Allele Prediction of MHC Class I-Presented Peptides by Incorporating Antigen Processing. Cell Syst.

[229] O'Donnell TJ, Rubinsteyn A, Bonsack M, Riemer AB, Laserson U, Hammerbacher J (2018). MHCflurry: Open-Source Class I MHC Binding Affinity Prediction. Cell Syst.

[230] Ong E, Cooke MF, Huffman A, Xiang Z, Wong MU, Wang H (2021). Vaxign2: the second generation of the first Web-based vaccine design program using reverse vaccinology and machine learning. Nucleic Acids Res.

[231] Ong E, Wang H, Wong MU, Seetharaman M, Valdez N, He Y (2020). Vaxign-ML: supervised machine learning reverse vaccinology model for improved prediction of bacterial protective antigens. Bioinformatics.

[232] He Y, Xiang Z, Mobley HL (2010). Vaxign: the first web-based vaccine design program for reverse vaccinology and applications for vaccine development. J Biomed Biotechnol.

[233] Salimi N, Fleri W, Peters B, Sette A (2012). The immune epitope database: a historical retrospective of the first decade. Immunology.

[234] Martini S, Nielsen M, Peters B, Sette A (2020). The Immune Epitope Database and Analysis Resource Program 2003-2018: reflections and outlook. Immunogenetics.

[235] Fleri W, Paul S, Dhanda SK, Mahajan S, Xu X, Peters B (2017). The Immune Epitope Database and Analysis Resource in Epitope Discovery and Synthetic Vaccine Design. Front Immunol.

[236] Martins J, Magalhães C, Rocha M, Osório NS (2019). Machine Learning-Enhanced T Cell Neoepitope Discovery for Immunotherapy Design. Cancer Inform.

[237] Borch A, Carri I, Reynisson B, Alvarez HMG, Munk KK, Montemurro A (2024). IMPROVE: a feature model to predict neoepitope immunogenicity through broad-scale validation of T-cell recognition. Front Immunol.

[238] Wongklaew P, Sriswasdi S, Chuangsuwanich E (2024). MHCSeqNet2-improved peptide-class I MHC binding prediction for alleles with low data. Bioinformatics.

[239] Lundegaard C, Lund O, Buus S, Nielsen M (2010). Major histocompatibility complex class I binding predictions as a tool in epitope discovery. Immunology.

[240] Wang M, Lei C, Wang J, Li Y, Li M (2024). TripHLApan: predicting HLA molecules binding peptides based on triple coding matrix and transfer learning. Brief Bioinform.

[241] Nilsson JB, Kaabinejadian S, Yari H, Kester MGD, van Balen P, Hildebrand WH (2023). Accurate prediction of HLA class II antigen presentation across all loci using tailored data acquisition and refined machine learning. Sci Adv.

[242] Reynisson B, Barra C, Kaabinejadian S, Hildebrand WH, Peters B, Nielsen M (2020). Improved Prediction of MHC II Antigen Presentation through Integration and Motif Deconvolution of Mass Spectrometry MHC Eluted Ligand Data. J Proteome Res.

[243] Karosiene E, Rasmussen M, Blicher T, Lund O, Buus S, Nielsen M (2013). NetMHCIIpan-3.0, a common pan-specific MHC class II prediction method including all three human MHC class II isotypes, HLA-DR, HLA-DP and HLA-DQ. Immunogenetics.

[244] Nielsen M, Lundegaard C, Blicher T, Lamberth K, Harndahl M, Justesen S (2007). NetMHCpan, a method for quantitative predictions of peptide binding to any HLA-A and -B locus protein of known sequence. PLoS One.

[245] Dhanda SK, Mahajan S, Manoharan M (2023). Neoepitopes prediction strategies: an integration of cancer genomics and immunoinformatics approaches. Brief Funct Genomics.

[246] Yalamarty SSK, Filipczak N, Li X, Subhan MA, Parveen F, Ataide JA (2023). Mechanisms of Resistance and Current Treatment Options for Glioblastoma Multiforme (GBM). Cancers (Basel).

[247] Shergalis A, Bankhead A 3rd, Luesakul U, Muangsin N, Neamati N (2018). Current Challenges and Opportunities in Treating Glioblastoma. Pharmacol Rev.

[248] Lin H, Liu C, Hu A, Zhang D, Yang H, Mao Y (2024). Understanding the immunosuppressive microenvironment of glioma: mechanistic insights and clinical perspectives. J Hematol Oncol.

[249] Rong L, Li N, Zhang Z (2022). Emerging therapies for glioblastoma: current state and future directions. J Exp Clin Cancer Res.

[250] Nabian N, Ghalehtaki R, Zeinalizadeh M, Balaña C, Jablonska PA (2024). State of the neoadjuvant therapy for glioblastoma multiforme-Where do we stand?. Neurooncol Adv.

[251] Lovly CM, Salama AK, Salgia R (2016). Tumor Heterogeneity and Therapeutic Resistance. Am Soc Clin Oncol Educ Book.

[252] Cheng L, Chen L, Shi Y, Gu W, Ding W, Zheng X (2024). Efficacy and safety of bispecific antibodies vs. immune checkpoint blockade combination therapy in cancer: a real-world comparison. Mol Cancer.

[253] Wang M, Zhang C, Wang X, Yu H, Zhang H, Xu J (2021). Tumor-treating fields (TTFields)-based cocktail therapy: a novel blueprint for glioblastoma treatment. Am J Cancer Res.

[254] Rios SA, Oyervides S, Uribe D, Reyes AM, Fanniel V, Vazquez J (2024). Emerging Therapies for Glioblastoma. Cancers (Basel).

[255] Fu M, Zhou Z, Huang X, Chen Z, Zhang L, Zhang J (2023). Use of Bevacizumab in recurrent glioblastoma: a scoping review and evidence map. BMC Cancer.

[256] Ghiaseddin A, Peters KB (2015). Use of bevacizumab in recurrent glioblastoma. CNS Oncol.

[257] Moser JC, Salvador E, Deniz K, Swanson K, Tuszynski J, Carlson KW (2022). The Mechanisms of Action of Tumor Treating Fields. Cancer Res.

[258] Morad G, Helmink BA, Sharma P, Wargo JA (2021). Hallmarks of response, resistance, and toxicity to immune checkpoint blockade. Cell.

[259] Catanzaro E, Beltrán-Visiedo M, Galluzzi L, Krysko DV (2025). Immunogenicity of cell death and cancer immunotherapy with immune checkpoint inhibitors. Cell Mol Immunol.

[260] Wang C, Yu M, Zhang W (2022). Neoantigen discovery and applications in glioblastoma: An immunotherapy perspective. Cancer Lett.

[261] Segura-Collar B, Hiller-Vallina S, de Dios O, Caamaño-Moreno M, Mondejar-Ruescas L, Sepulveda-Sanchez JM (2023). Advanced immunotherapies for glioblastoma: tumor neoantigen vaccines in combination with immunomodulators. Acta Neuropathol Commun.

[262] Tapescu I, Madsen PJ, Lowenstein PR, Castro MG, Bagley SJ, Fan Y (2024). The transformative potential of mRNA vaccines for glioblastoma and human cancer: technological advances and translation to clinical trials. Front Oncol.

[263] Strika Z, Petković K, Likić R (2024). Effectiveness and Safety of mRNA Vaccines in the Therapy of Glioblastoma. J Pers Med.

[264] Restifo NP, Dudley ME, Rosenberg SA (2012). Adoptive immunotherapy for cancer: harnessing the T cell response. Nat Rev Immunol.

[265] Dagar G, Gupta A, Masoodi T, Nisar S, Merhi M, Hashem S (2023). Harnessing the potential of CAR-T cell therapy: progress, challenges, and future directions in hematological and solid tumor treatments. J Transl Med.

[266] Deng Z, Zhan P, Yang K, Liu L, Liu J, Gao W (2022). Identification of personalized neoantigen-based vaccines and immune subtype characteristic analysis of glioblastoma based on abnormal alternative splicing. Am J Cancer Res.

[267] Zhang X, Zhao L, Zhang H, Zhang Y, Ju H, Wang X (2022). The immunosuppressive microenvironment and immunotherapy in human glioblastoma. Front Immunol.

[268] Bell M, Gottschalk S (2021). Engineered Cytokine Signaling to Improve CAR T Cell Effector Function. Front Immunol.

[269] Muhammad S, Fan T, Hai Y, Gao Y, He J (2023). Reigniting hope in cancer treatment: the promise and pitfalls of IL-2 and IL-2R targeting strategies. Mol Cancer.

[270] Wei F, Cheng XX, Xue JZ, Xue SA (2022). Emerging Strategies in TCR-Engineered T Cells. Front Immunol.

[271] Baulu E, Gardet C, Chuvin N, Depil S (2023). TCR-engineered T cell therapy in solid tumors: State of the art and perspectives. Sci Adv.

[272] McGrath K, Dotti G (2021). Combining Oncolytic Viruses with Chimeric Antigen Receptor T Cell Therapy. Hum Gene Ther.

[273] Luksik AS, Yazigi E, Shah P, Jackson CM (2023). CAR T Cell Therapy in Glioblastoma: Overcoming Challenges Related to Antigen Expression. Cancers (Basel).

[274] Kringel R, Lamszus K, Mohme M (2023). Chimeric Antigen Receptor T Cells in Glioblastoma-Current Concepts and Promising Future. Cells.

[275] Bartoszewska E, Tota M, Kisielewska M, Skowron I, Sebastianka K, Stefaniak O (2024). Overcoming Antigen Escape and T-Cell Exhaustion in CAR-T Therapy for Leukemia. Cells.

[276] Lin H, Yang X, Ye S, Huang L, Mu W (2024). Antigen escape in CAR-T cell therapy: Mechanisms and overcoming strategies. Biomed Pharmacother.

[277] Grosser R, Cherkassky L, Chintala N, Adusumilli PS (2019). Combination Immunotherapy with CAR T Cells and Checkpoint Blockade for the Treatment of Solid Tumors. Cancer Cell.

[278] Sharma P, Goswami S, Raychaudhuri D, Siddiqui BA, Singh P, Nagarajan A (2023). Immune checkpoint therapy-current perspectives and future directions. Cell.

[279] Ou X, Ma Q, Yin W, Ma X, He Z (2021). CRISPR/Cas9 Gene-Editing in Cancer Immunotherapy: Promoting the Present Revolution in Cancer Therapy and Exploring More. Front Cell Dev Biol.

[280] Chen X, Zhong S, Zhan Y, Zhang X (2024). CRISPR-Cas9 applications in T cells and adoptive T cell therapies. Cell Mol Biol Lett.

[281] Stephens AJ, Burgess-Brown NA, Jiang S (2021). Beyond Just Peptide Antigens: The Complex World of Peptide-Based Cancer Vaccines. Front Immunol.

[282] Kanaly CW, Ding D, Heimberger AB, Sampson JH (2010). Clinical applications of a peptide-based vaccine for glioblastoma. Neurosurg Clin N Am.

[283] Noushmehr H, Herrgott G, Morosini NS, Castro AV (2022). Noninvasive approaches to detect methylation-based markers to monitor gliomas. Neurooncol Adv.

[284] García-Montaño LA, Licón-Muñoz Y, Martinez FJ, Keddari YR, Ziemke MK, Chohan MO (2023). Dissecting Intra-tumor Heterogeneity in the Glioblastoma Microenvironment Using Fluorescence-Guided Multiple Sampling. Mol Cancer Res.

[285] Lv X, Wang B, Liu K, Li MJ, Yi X, Wu X (2024). Decoding heterogeneous and coordinated tissue architecture in glioblastoma using spatial transcriptomics. iScience.

[286] Jacquemin V, Antoine M, Dom G, Detours V, Maenhaut C, Dumont JE (2022). Dynamic Cancer Cell Heterogeneity: Diagnostic and Therapeutic Implications. Cancers (Basel).

[287] Yabo YA, Niclou SP, Golebiewska A (2022). Cancer cell heterogeneity and plasticity: A paradigm shift in glioblastoma. Neuro Oncol.

[288] Lindau D, Gielen P, Kroesen M, Wesseling P, Adema GJ (2013). The immunosuppressive tumour network: myeloid-derived suppressor cells, regulatory T cells and natural killer T cells. Immunology.

[289] Haist M, Stege H, Grabbe S, Bros M (2021). The Functional Crosstalk between Myeloid-Derived Suppressor Cells and Regulatory T Cells within the Immunosuppressive Tumor Microenvironment. Cancers (Basel).

[290] Gonzalez H, Hagerling C, Werb Z (2018). Roles of the immune system in cancer: from tumor initiation to metastatic progression. Genes Dev.

[291] Motz GT, Coukos G (2013). Deciphering and reversing tumor immune suppression. Immunity.

[292] Kim SK, Cho SW (2022). The Evasion Mechanisms of Cancer Immunity and Drug Intervention in the Tumor Microenvironment. Front Pharmacol.

[293] Cui JW, Li Y, Yang Y, Yang HK, Dong JM, Xiao ZH (2024). Tumor immunotherapy resistance: Revealing the mechanism of PD-1 / PD-L1-mediated tumor immune escape. Biomed Pharmacother.

[294] Lin X, Kang K, Chen P, Zeng Z, Li G, Xiong W (2024). Regulatory mechanisms of PD-1/PD-L1 in cancers. Mol Cancer.

[295] Seidel JA, Otsuka A, Kabashima K (2018). Anti-PD-1 and Anti-CTLA-4 Therapies in Cancer: Mechanisms of Action, Efficacy, and Limitations. Front Oncol.

[296] Shang S, Zhao Y, Qian K, Qin Y, Zhang X, Li T (2022). The role of neoantigens in tumor immunotherapy. Biomed Pharmacother.

[297] Qi Y, Zhang L, Liu Y, Li Y, Liu Y, Zhang Z (2024). Targeted modulation of myeloid-derived suppressor cells in the tumor microenvironment: Implications for cancer therapy. Biomed Pharmacother.

[298] Rossetti R, Brand H, Lima SCG, Furtado IP, Silveira RM, Fantacini DMC (2022). Combination of genetically engineered T cells and immune checkpoint blockade for the treatment of cancer. Immunother Adv.

[299] Khair DO, Bax HJ, Mele S, Crescioli S, Pellizzari G, Khiabany A (2019). Combining Immune Checkpoint Inhibitors: Established and Emerging Targets and Strategies to Improve Outcomes in Melanoma. Front Immunol.

[300] Vafaei S, Zekiy AO, Khanamir RA, Zaman BA, Ghayourvahdat A, Azimizonuzi H (2022). Combination therapy with immune checkpoint inhibitors (ICIs); a new frontier. Cancer Cell Int.

[301] Yu G, He X, Li X, Wu Y (2022). Driving neoantigen-based cancer vaccines for personalized immunotherapy into clinic: A burdensome journey to promising land. Biomed Pharmacother.

[302] Ni L (2023). Advances in mRNA-Based Cancer Vaccines. Vaccines (Basel).

[303] Li C, Jiang P, Wei S, Xu X, Wang J (2020). Regulatory T cells in tumor microenvironment: new mechanisms, potential therapeutic strategies and future prospects. Mol Cancer.

[304] Chen Y, Zhou Q, Jia Z, Cheng N, Zhang S, Chen W (2024). Enhancing cancer immunotherapy: Nanotechnology-mediated immunotherapy overcoming immunosuppression. Acta Pharm Sin B.

[305] Supabphol S, Li L, Goedegebuure SP, Gillanders WE (2021). Neoantigen vaccine platforms in clinical development: understanding the future of personalized immunotherapy. Expert Opin Investig Drugs.

[306] Kumari K, Singh A, Chaudhary A, Singh RK, Shanker A, Kumar V (2024). Neoantigen Identification and Dendritic Cell-Based Vaccines for Lung Cancer Immunotherapy. Vaccines (Basel).

[307] Jogalekar MP, Rajendran RL, Khan F, Dmello C, Gangadaran P, Ahn BC (2022). CAR T-Cell-Based gene therapy for cancers: new perspectives, challenges, and clinical developments. Front Immunol.

[308] Zappasodi R, Merghoub T, Wolchok JD (2018). Emerging Concepts for Immune Checkpoint Blockade-Based Combination Therapies. Cancer Cell.

[309] Ren L, Leisegang M, Deng B, Matsuda T, Kiyotani K, Kato T (2019). Identification of neoantigen-specific T cells and their targets: implications for immunotherapy of head and neck squamous cell carcinoma. Oncoimmunology.

[310] Kula T, Dezfulian MH, Wang CI, Abdelfattah NS, Hartman ZC, Wucherpfennig KW (2019). T-Scan: A Genome-wide Method for the Systematic Discovery of T Cell Epitopes. Cell.

[311] Dezfulian MH, Kula T, Pranzatelli T, Kamitaki N, Meng Q, Khatri B (2023). TScan-II: A genome-scale platform for the de novo identification of CD4^+^ T cell epitopes. Cell.

[312] Pearlman AH, Hwang MS, Konig MF, Hsiue EH, Douglass J, DiNapoli SR (2021). Targeting public neoantigens for cancer immunotherapy. Nat Cancer.

[313] Liu B, Zhou H, Tan L, Siu KTH, Guan XY (2024). Exploring treatment options in cancer: Tumor treatment strategies. Signal Transduct Target Ther.

[314] Birnboim-Perach R, Benhar I (2024). Using Combination therapy to overcome diverse challenges of Immune Checkpoint Inhibitors treatment. Int J Biol Sci.

[315] Cheng AL, Hsu C, Chan SL, Choo SP, Kudo M (2020). Challenges of combination therapy with immune checkpoint inhibitors for hepatocellular carcinoma. J Hepatol.

[316] Ruan S, Greenberg Z, Pan X, Zhuang P, Erwin N, He M (2022). Extracellular Vesicles as an Advanced Delivery Biomaterial for Precision Cancer Immunotherapy. Adv Healthc Mater.

[317] Wang L, Yu X, Zhou J, Su C (2023). Extracellular Vesicles for Drug Delivery in Cancer Treatment. Biol Proced Online.

